# Medical students’ perception of changes in assessments implemented during the COVID-19 pandemic

**DOI:** 10.1186/s12909-022-03787-9

**Published:** 2022-12-07

**Authors:** Francesca Bladt, Prakriti Khanal, Anusha Mahesh Prabhu, Elizabeth Hauke, Martyn Kingsbury, Sohag Nafis Saleh

**Affiliations:** 1 Imperial College School of Medicine, Imperial College London, London, UK; 2 Centre for Languages, Culture, and Communication, Imperial College London, London, UK; 3 Centre for Higher Education Research, Imperial College London, London, UK

**Keywords:** COVID-19, Assessment, Medical education, Examinations, Online examination, Open-book, Supervised, Medical students, Proctoring

## Abstract

**Background:**

COVID-19 posed many challenges to medical education in the United Kingdom (UK). This includes implementing assessments during 4 months of national lockdowns within a 2-year period, where in-person education was prohibited. This study aimed to identify medical school assessment formats emerging during COVID-19 restrictions, investigate medical students’ perspectives on these and identify influencing factors.

**Methods:**

The study consisted of two phases: a questionnaire asking medical students about assessment changes they experienced, satisfaction with these changes and preference regarding different assessments that emerged. The second phase involved semi-structured interviews with medical students across the UK to provide a deeper contextualized understanding of the complex factors influencing their perspectives.

**Results:**

In the questionnaire responses, open-book assessments had the highest satisfaction, and were the preferred option indicated. Furthermore, in the case of assessment cancellation, an increase in weighting of future assessments was preferred over increase in weighting of past assessments. Students were also satisfied with formative or pass-fail assessments.

Interview analyses indicate that although cancellation or replacement of summative assessments with formative assessments reduced heightened anxiety from additional COVID-19 stressors, students worried about possible future knowledge gaps resulting from reduced motivation for assessment-related study. Students’ satisfaction level was also affected by timeliness of communication from universities regarding changes, and student involvement in the decision-making processes. Perceived fairness and standardisation of test-taking conditions were ranked as the most important factors influencing student satisfaction, followed closely by familiarity with the format. In contrast, technical issues, lack of transparency about changes, perceived unfairness around invigilation, and uncertainty around changes in assessment format and weighting contributed to dissatisfaction.

**Conclusions:**

Online open-book assessments were seen as the most ideal amongst all participants, and students who experienced these were the most satisfied with their assessment change. They were perceived as most fair and authentic compared to real-life medical training. We seek to inform educators about student perceptions of successful assessment strategies under COVID-19 restrictions and provide evidence to allow debate on ongoing assessment reform and innovation. While this work looks specifically at assessment changes during COVID-19, understanding factors affecting student perception of assessment is applicable to examinations beyond COVID-19.

**Supplementary Information:**

The online version contains supplementary material available at 10.1186/s12909-022-03787-9.

## Introduction

Written assessments, alongside practical clinical assessments, are a key part of UK medical school curricula. They ensure that students meet the requisite standards for clinical competence and have the knowledge requirements necessary to become a proficient doctor [1, 2].

Assessments can be formative and/or summative in nature. Formative assessment (assessment as a motivation for learning) primarily offers feedback to build knowledge and skills while summative assessment (assessment of learning) tends to be criterion referenced and is ultimately more about evaluating whether a student has reached the pre-defined General Medical Council criteria required of a safe doctor, to reassure the public [3, 4]. Furthermore, assessments tend to drive learning and help medical students to prioritise particular aspects of the extensive curriculum [3, 5, 6]. Therefore, any changes to assessment methods may have a direct influence on learning and also impact the knowledge and skills of future doctors [1, 3, 5, 6].

Medical school assessments have traditionally taken place with in-person attendance at a controlled and invigilated space; preventing collusion and cheating, providing a quiet and standardised test taking environments for all students, and ensuring assessment is completed at a single timepoint [7–10]. The methods of assessment have shifted over time from essay-based assessment to multiple choice tests, due to concerns about the small sample of knowledge tested and subjectivity of marking between examiners [3]. Other important aspects such as clinical skills, attitude and communication are tested with practical assessments such as objective structured clinical examinations (OSCEs) and practical assessment of clinical examination skills (PACES). [3].

The unprecedented circumstances of the COVID-19 pandemic meant that traditional invigilated closed-book assessments were no longer possible. Thus, alternative assessment formats, such as online open-book examination (OBE) or coursework-based assessment, had to be introduced, with higher education institutes (HEIs) implementing a wide variety of strategies and formats. It is important to learn from the 2020 summer assessment period to see which strategies worked well and what benefits remote assessments provided [8, 11, 12]. Assessments implemented during COVID-19 included: online open or closed-book examinations, examination cancellation with increased weighting of other assessments, or coursework-based assessment. There are currently contradictory findings in the literature over how the discriminative ability of OBEs compares to closed-book examinations (CBE) traditionally used during in-person invigilated assessment [13–21]. While some studies have shown comparable student performance [13–16], pass rates [20] and ability to discriminate in OBE and CBE, other research demonstrates CBEs are superior to OBEs in these respects [17] and vice versa [21]. Even when the cohort as a whole show similar performances in both assessment scenarios, it is impossible to demonstrate whether the performances of each individual have stayed at the same levels [22, 23]. This would be particularly important to consider for high-stakes assessments. The creation of difficult circumstances and exacerbation of pre-existing challenges for some students during COVID-19, such as limited access to technology or illness, makes this issue even more pertinent and further research is required in this area [8].

It is also important to consider student preferences, to minimise additional student distress, anxiety, and dissatisfaction, and ensure students feel well equipped for future career progression. The assessments during this period largely had to be implemented in a short amount of time with minimal student consultation. Students sat and prepared for assessments amidst great institutional, logistical, social and personal disruption and uncertainty, and this likely impacted their emotional preparedness and confidence [8].

Therefore, the aim of this study is to identify and categorise the range of changes in assessment formats that have emerged during the COVID-19 pandemic, as well as investigate medical students’ perspectives on them and the reasons contributing to these views. The data collected can hopefully be used by medical educators to understand students’ views on their preferred assessment format when it is unadvisable to implement traditional assessments and to understand medical student perceptions of assessment in general.

## Methods

This study was composed of two parts: the first part being a questionnaire disseminated to medical students across the UK, and the second part being a series of one-on-one interviews. This study obtained ethical approval for data collection and analysis from the Imperial College London Education Ethics Review Process (EERP1920-104) and was aligned with good ethical practice and BERA guidelines. All methods and analyses were carried out in accordance with the approved guidelines and regulations. Participants were medical students based in the UK (studying MBBS/MBChB/BMBS/MB BChir/BM BCh or equivalent courses) that had a written assessment scheduled between 01/03/2020 and 31/07/2020. No data was captured on practical clinical skills examinations such as OSCE or PACES.

### Questionnaire

Recruitment for this cross-sectional study was conducted via various medical student chat rooms (such as The Student Room, and Facebook medical student group chats) and via e-mailing medical student societies at all UK universities offering medicine courses. The questionnaire was delivered online using the Qualtrics® platform and consisted of a combination of multiple-choice questions, Likert scale questions, and open-ended free-text questions (see Table 1). The questionnaire was designed to allow response-dependent branching, which meant participants were not required to complete any redundant questions. The statistical analysis software, GraphPad Prism (version 8) was used for quantitative data analysis and figure production.

**Table 1:** Questionnaire

What was your year of study in the academic year 2019–2020?	•Pre-clinical •Clinical •Intercalated
Were there any factors that impacted your assessment experience? •(e.g., time zone (being overseas), travel, access to quiet areas to study/take assessments, access to resources, special learning arrangements, COVID-19 related mitigating circumstances)	
Did your exam format change due to the Covid-19 crisis?	•Yes •No
What was the format of your written exams during the COVID -19 Crisis? (If multiple exams were affected, select all that apply) How satisfied were you with these changes and why?	•Cancelled exam with previous exams having increased contribution towards your degree •Cancelled exam with future exams having increased contribution towards your degree •Postponed exam with another date •Essay or coursework-based assessment •Completing your exam online at home unsupervised but not allowed to look at notes •Completing your exam online at home while being supervised and not allowed to look at notes •Completing your exam online at home and allowed to look at notes •Other (Please specify)
How did your examination contribution towards your degree change during the COVID-19 Crisis? (If multiple exams were affected, select multiple if they apply)	•Changed to pass/fail •Changed to summative + prizes (Merit/distinction/distinction*) •Changed to summative only •Changed to formative •Changed in percentage weighting •Contribution did not change •Other
How satisfied were you with these changes?	•Likert scale – 1–5 (for each option selected)
Why?	[free text]
Rank the examination styles from most preferred to least preferred. (Drag and drop to reorder the options)	1.Completing your exam online at home and allowed to look at notes 2.Completing your exam online at home while being supervised and not allowed to look at notes 3.Completing your exam online at home unsupervised but not allowed to look at notes 4.Postpone exam to in hall assessment 5.Essay or coursework-based assessment 6.Cancelled exam with future exams having increased contribution towards your degree •Cancelled exam with previous exams having increased contribution towards your degree
Why was your most preferred option your most preferred option?	1[free text]
Why was your least preferred option your least preferred option?	2[free text]
Which of these aspects would enhance your satisfaction with changes to the exam? Please rank them in order of importance. (Drag and drop to reorder the options)	1.Familiarity of the exam format 2.Change in contribution of exams towards your degree 3.Fairness (e.g., being supervised, limiting colluding with others) 4.Exam environment (being at home, being able to eat/ drink, being in a hall) 5.Convenience of exam (e.g., able to highlight/ flag, access to Wi-Fi/technology, open-book) 6.Convenient timing of exam

(Table 1) shows the questions asked to participants to gather information on the assessment formats they experienced and viewpoints on these

### Interviews

Interview participants were selected from questionnaire participants who had consented to be approached by providing contact details at the end of the questionnaire. Entry into a prize draw for a £40 voucher was offered to participants who took part in the interview. All participants interested in the interview were contacted via e-mail to participate in an online semi-structured interview conducted via Microsoft Teams.

The semi-structured interview was scheduled for 30 min and covered questions about the assessment changes experienced by students and their feelings about these, the relative importance of different aspects that affected their assessments, their ideal assessment and their confidence going into the assessment.

Thematic analysis of the interview transcripts as outlined by Braun & Clarke [24], was used to capture important and recurrent themes using coding. To increase the dependability and generalisability of the final themes/sub-themes, analysis was carried out by 4 team members independently and was then discussed during several meetings and reassembled into the final codes for analysis. During these meetings, any additional themes/sub-themes were added where necessary and disagreements were resolved through discussion. Each theme/sub-theme identified was then checked again in relation to their frequency within each interview transcript.

Table 1 shows the questions asked to participants within the questionnaire phase of this study to collect information on the assessment formats students experienced and their viewpoints on these.

## Results

### Medical student characteristics

Medical students who participated in the survey (*n* = 119) came from 21 different universities across the UK. There were comparable numbers of students at pre-clinical stages (48.7%, *n* = 58) and in the clinical years (47.1%, *n* = 56) of their studies. The remaining students classified themselves as intercalating (4.2%, *n* = 5).

Questionnaire participants who had consented to be approached (*n* = 51) were contacted via e-mail. Due to schedule availability and loss to follow up, *n* = 6 participants proceeded with the interview. The participants were either in their clinical years (*n* = 3) or pre-clinical years (*n* = 3).

### Frequency and Satisfaction of Interventions

**Fig. 1:**
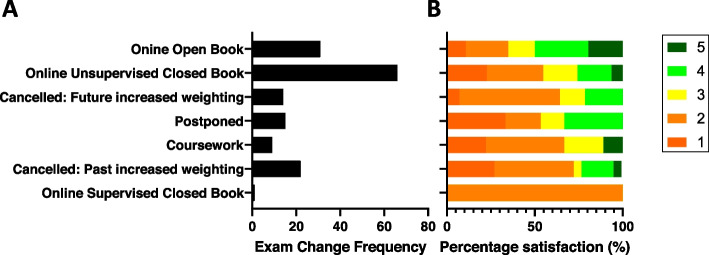
Frequency of assessment format changes and satisfaction with changes. The frequency of the assessment weighting changes (**A**) and satisfaction amongst those experiencing each assessment weighting change (**B**) during the COVID-19 pandemic in 119 medical students were plotted in a bar graph in order of satisfaction from most popular at the top, to least popular at the bottom. **A**. The bar graph depicts the number of medical students that had each change in weighting. **B**. The bar graph shows the percentage satisfaction with a certain weighting change with 5 (dark green) = extremely satisfied, 4 (light green) = satisfied, 3 (yellow) = neutral, 2 (orange) = unsatisfied and 1(red) = extremely unsatisfied

**Fig. 2:**
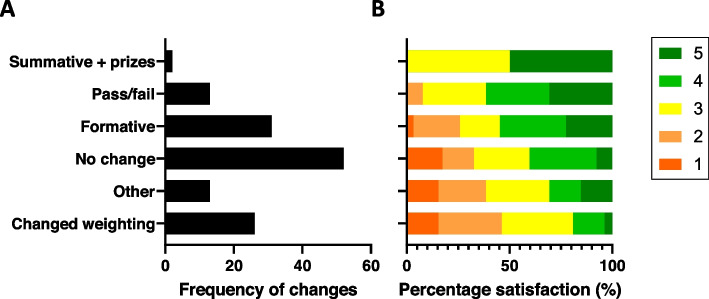
Frequency of assessment weighting changes and satisfaction with these changes. The frequency of the assessment format changes (**A**) and satisfaction amongst those experiencing each format change (**B**) during the COVID-19 pandemic in 119 medical students were plotted in a bar graph in order of satisfaction from most popular at the top to least popular at the bottom. **A** The bar graph depicts the number of medical students that experienced each assessment change. **B** The bar graph shows the percentage satisfaction with each particular assessment change with 5 (dark green) = extremely satisfied, 4 (light green) = satisfied, 3 (yellow) = neutral, 2 (orange) = unsatisfied and 1 (red) = extremely unsatisfied.

We analysed the questionnaire data on the different types of assessments implemented during the COVID-19 pandemic and student satisfaction with these (Fig. 1 and 2). The three most commonly implemented assessment changes were: online unsupervised CBEs (41.7%), online OBEs (19.6%) and cancellation of assessment with increased weighting of previous assessment (13.9%) (Fig. 1A). Furthermore, the assessment changes with the highest satisfaction rating were online OBEs, followed by online unsupervised CBEs, and then cancellation of assessment with increased weighting of future assessments (Fig. 1B). The least preferred option was the online supervised CBEs, although only one survey participant experienced this change.

In terms of assessment weighting changes, the three most common changes were: no changes (37.9%), making assessment formative (22.6%) and a change in weighting of other assessments (18.9%) (Fig. 2A). Furthermore, the assessment weighting changes that had the highest satisfaction ratings were: change to summative assessment with prizes, change to pass/fail and making assessments formative (Fig. 2B).

### Preferred assessment change and factors impacting satisfaction

**Fig. 3:**
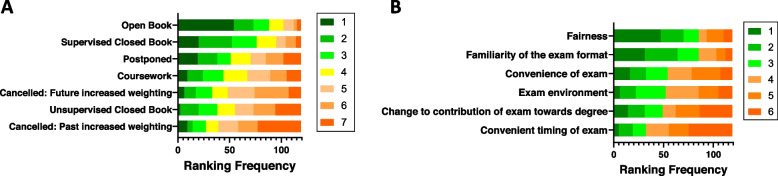
Highest ranked, ideal assessment formats and features of assessment that affect satisfaction. The bar graph depicts these in order from most popular first to least popular last. **A** The bar graph depicts the ranking by medical students of their most preferred [1] to least preferred [7] assessment format. **B** The bar graph depicts the ranking by medical students of factors of an assessment that are most important in affecting their satisfaction [1], to least important [6]

Next, to identify which assessment format would have been most preferred and what factors impacted student satisfaction, medical students were asked to rank several options. OBEs, supervised CBEs, and postponed assessment were the assessment changes that medical students would have ideally preferred during the COVID-19 pandemic (Fig. 3A). In terms of factors that impacted student satisfaction, the most important were fairness, familiarity with the format, and convenience of the assessment (Fig. 3B).

Assessment format preference amongst all participants during the COVID-19 pandemic and the factors impacting satisfaction with assessment were ranked from most preferred/important [1] to least [7] by medical students(*n* = 119).

**Fig. 4:**
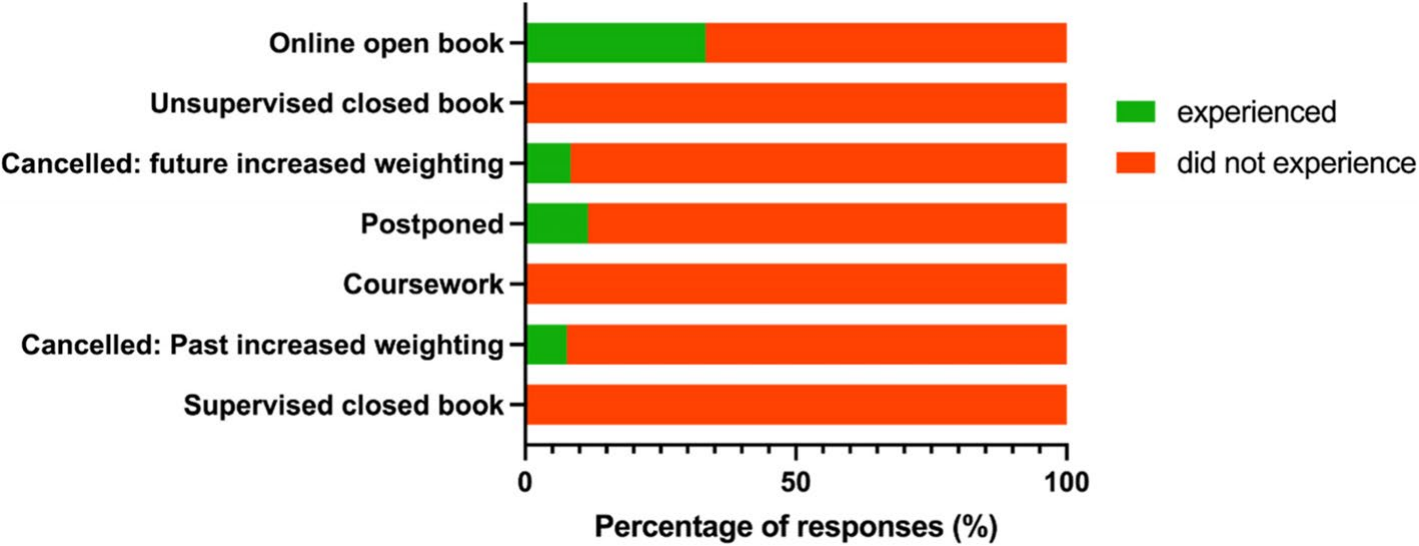
Proportion of students experiencing their preferred assessment format in the 2020 summer assessment period

We further explored whether students’ most preferred assessment format was affected by whether they had actually experienced that assessment. We found that for most assessment formats, a very small proportion had actually experienced their preferred assessment format during the 2020 summer assessment period (Fig. 4). However, out of those who preferred online OBEs, which was the highest ranked assessment format, a much larger proportion (33.3%) of students had experienced this in the 2020 summer assessment period (Fig. 4). It is important to note that several students experienced more than one assessment format.

The percentage of students that experienced (green) and did not experience (red) their preferred assessment format during COVID-19 in 119 medical students were plotted in a bar graph according to the different assessment formats they experienced. The number of responses of assessment formats experienced is *n* = 148, since some students experienced multiple assessment formats.

In open text questions, students described a variety of personal circumstances that affected their assessment performance, including from most to least common: lack of access to quiet study areas (*n* = 31), lack of access to usual study resources and libraries (*n* = 14), internet access difficulties(*n* = 13), family circumstances (*n* = 11), being in different time zones overseas (*n* = 7), social isolation/needing to shield and resulting poor mental health (*n* = 5), lack of teaching during lockdown (*n* = 4), challenges with motivation in a home environment (*n* = 2), working as essential workers during COVID-19 (*n* = 1), and financial issues (*n* = 1).

### Interview analysis

Thematic analysis was used to analyse interview transcripts from *n* = 6 participants. The three main themes discovered were: sufficient and timely communication from the HEI, impact on preparation for the assessment, and feelings about the assessment format and weighting. The themes were then divided into twelve different sub-themes (see Table 2). The most common theme found within the transcripts was feelings about the assessment changes, which contains eight sub-themes within it. The subthemes of ‘uncertainty’ and ‘technical issues’ were the most common as they were found in 100% of the transcripts. The sub-themes of ‘decision making’, ‘personal circumstances’, and ‘postponement’ were the least mentioned and found in only 33% of the transcripts. Though we did not look at the effect of online teaching, some students also reported that lack of in-person teaching made them feel less prepared, as expressed by this quote from transcript 1:

“We moved to online teaching. So, for the last three or four weeks, I felt as if I didn't learn as much as I probably should have in terms of content for my exam"

**Table 2:** Thematic analysis of the interviews

Theme	Subtheme	Representative quote (transcript number)
**Communication**	Uncertainty—including transparency & timelines	"I'd be much happier being prepared even if it was one or two weeks in advance about whether it is an online exam or in person exam and if its long writing or multiple-choice answer question. Just having a knowledge of the format of the exam would help" (1)
		"I'm actually not entirely sure how they have changed because I'm intercalating right now, and I can't remember if they told us. Actually, no they did" (2)
		"There’s just so many uncertainties of how they're going to weigh up the deciles… med schools still haven't really gave[sic] us quite a lot of information … so I don’t really know what's going to happen" (3)
		"We didn’t know when our exam was, we didn't know the time, we didn't know the format and we were just informed a week before" "We don't know the system, and… we find ourselves simply saying yes, ok, we agree, we don't have a choice, let's go along with it" (4)
		"It would be nice if the faculty could be a bit clearer on the sort of future and where this is going (…)I guess it is annoying as a student that you are kind of in the blind a little bit, but I guess I'll just be patient and wait " (5)
		“Oh, I will have to check that very quickly” (6)
	Decision-making	"I'm glad that they're doing like a kind of mass voting system, so as a cohort we kind of can decide what happens as opposed to just med school deciding for us". (3)
		“I think it’s important…- firstly, the idea of it being democratic, so up to the students to decide… was very smart” (4)
**Preparation**	Personal circumstances	"I was one of those people that was basically working full-time in hospitals helping out- I found it quite challenging trying to manage my time" "I live with my sister and we’re very close, so she disturbs me all the time. So that’s a big distracting factor" (3)
		"My mental health just deteriorated really, in lockdown" "It got to a point where I haven't had my laptop for about two months, three months, and I've been really ill, so, in terms of confidence—there was none if I'm honest… we got evicted as well" (4)
	Unfamiliarity/ familiarity	"I think it was my lack of practice of these open-book exams that made me feel like I didn't have as much time as I may have had" (1)
		"Aspect of it not being consistent with previous exams, with the aspect of not having mock papers to utilize" “Nothing like what our mock papers were like…much, much harder” (4)
		"I was slightly unsure, because not many exams have been done online previously" (5)
		"Probably understanding the format of the exam, kind of being familiar with it. I was familiar with it, but if they'd all of a sudden said oh, it's going to be like essays, I'd be like confused” (6)
**Assessment**	Change to formative	"So I didn't have that anxiety related to the exam, but at the same time, I didn't learn anything for this exam like I didn't study properly because I had already known it was a formative" "Because it's a formative it can't count towards like FPAS applications or ranking, and a lot of people were banking on these exams to improve their grade or just improve their ranking" (2)
		"I think if the exam was how I would like it to be, which would be online open-book, then I probably would quite like it to be a summative exam. Just so that it counts for something?" (3)
		"I would have preferred there wasn't an exam if I'm honest or if there was an exam it was purely formative to gauge where we were at" (4)
	Cancellation	"It was nice to not have to worry about that alongside your main exams as well as anything else that you're doing" (1)
		“I would rather have future exams having more influence than previous exams because I can't change my previous results” (2)
		"But going into third year, I probably would say that my confidence isn’t as great because I haven’t been tested on the knowledge, I don't know if I've learned everything" "I can imagine if you've been studying for a while and then it’s been cancelled that would be quite annoying" (3)
		"because of the OSCE being cancelled. I personally put more focus on the other exams and- so it does make me slightly worried for example, when I reach my second clinical year, which is in fifth year, will it be difficult for me to pick up these things " (5)
		"So the fact it was cancelled gave me more time to just focus solely on the exam. And obviously it was a bit of a stressful time for everyone" (6)
	Change to format—online/open-book	"Having access to notes was less beneficial than I would have thought so I think based on the content and the timing of the exam it was a lot more difficult" "it's better that they sort of allowed it to be open book because I think that's a more understanding approach than universities or exams and courses, which sort of used eye tracking software and had people watch over you while you're taking your exams. " (1)
		"If it's online, like you said, it can't be supervised like an in-person exam, so a lot of people might look through their notes or Google things then in which will be unfair… even if it’s supervised or unsupervised and open book (…) there will be a few people that would act as if it's open book anyway. So, I would prefer if it's unsupervised, just because I think if it is supervised then it'll get a bit complicated in terms of how it would work "(3)
		"So I think the idea of it being open book and online is the way forward (…) for the ease of marking and data protection and just the collation of data in terms of ranking, but also in terms of, you could say, for the environment as well as other things so the idea of things being online isn’t a new aspect, the aspect of it being open book is very new to us students." "In light of the pandemic they made the exam harder in order to compensate for the open book aspect" (4)
		"I knew if I didn't learn the content in this online exam then I could just find it in my notes"(5)
		"I was a bit more confident as I knew it was open book, so I knew I always had something to rely on, like my notes"(6)
	Postponement	"they've moved the 4th year exams into a month where they're going to have their finals so that's going to be a really really stressful month for these students"(4)
		we've never done like an examined OSCE before, so that will be the first time in Year 4 which is, I mean, that's quite far into medical school so yeah, I mean, I'll just take it as it comes” (6)
	Technical issues	"If the screen brightness is a bit low that could severely impact your ability to understand what the stuff you’re looking at " (1)
		"I think in quarantine everyone had like difficulty with Wi-Fi, so in that circumstance I would not like the pressure of having like ‘Okay your papers open for two hours’" (2)
		"Like really bad Wi-Fi on your laptop and things like that may prevent you from doing it" (3)
		"a lot of my friends elsewhere within the UK or abroad had poorer Internet connections than they did when they were at university for example… I'd expect them [the university] to obviously run diagnostics, to ensure their software is reliable, to ensure that someone's on hand to, to answer any questions " (4)
		"I guess technical problems like, if you know that you have a really dodgy Internet connection, then you could be quite worried on the day of your exam " (5)
		"Like I think I would have preferred that they've given us a phone number, actually, if I had technical issues and stuff” (6)
	Environment	"I've completed exams in noisy environments, quiet environments. I’ve completed exams outside, inside. Haven't really seen too much of a difference" (1)
		"I didn't mind sitting at home because, my family were understanding, and I was comfortable in my own room, and I was able to like sit the paper and I had like snacks and food and water" (2)
		"Taking an exam for 4 h for my partners was quite disrupted for her because she was working from home on the Internet on her computer"(4)
		"Having the home environment as well, it was familiar so that was nice " "The fact that students had different home environments or perhaps different residential environments where they carried out the exam could then perhaps could have led to slight disparities, which may mean that their performance was affected by the environment" (5)
	Fairness/ standardisation	"For people who were overseas and had different time zones…so it was like midday to help them"(1)
		" It was open for two days, like time difference- because a few of my friends are international I would have been worried for them if like it was only open for like 2 h and they had such a big-time difference"(2)
		"Everyone is kind of in the same condition, doing it at the same time and being all supervised. There's no kind of variable in that"(3)
		"don’t see any benefit from examining students in the middle of pandemic when they can't allow an equal playing field for the numerous people- accounting for their countries, given personal circumstances and the impact that it's had on their personal lives… there should be an option that someone should be able to go in halls " (4)
		"You want the exam to be under a controlled environment in order to make it fair and representative and so therefore, in person exams in an exam hall etc. would allow that, sort of a, control element" "Obviously an environment that's controlled so there's no sort of cheating and things"(5)
	Applicability to practice	"I would prefer like a clinical case and then ask questions from there because it's more like what we would be experiencing in real life "(2)
		"don't really like it when they ask you like specific questions on specific diseases or medication that you just memorise, but you might not actually use that information later-on" (3)
		"Open book and unsupervised, as I think more accurately, perhaps it represents what we will be experiencing as F1/F2 doctors in a clinical setting—aspects of reliance and interdisciplinary decision-making as an MDT" (4)
		“Bring in the more practical aspects of what we do and shun maybe more of the theoretical, non-practical, dare I say, less useful aspects instead” (6)

The table shows the thematic analysis of 6 transcripts from interviews with 6 different participants. It is composed of three different sections which corresponds to the three main themes of the transcripts. Each theme is divided into different sub-themes. There is one representative quote from each transcript which contains that particular sub-theme. Extra quotes under some sub-themes cover an aspect of the sub-theme not covered by the other quotes

Table 2 shows the thematic analysis of six transcripts from interviews with six different participants. It is composed of three different sections which corresponds to the three main themes of the transcripts. Each theme is divided into different sub-themes. In each sub-theme section, one representative quote from each transcript containing that particular sub-theme has been included- the transcript number is indicated within brackets after each quote. Multiple quotes from the same transcript were included under some sub-themes if they covered an aspect of the sub-theme not covered by the other quotes from other transcripts.

## Discussion

This mixed-methods national study explored medical students’ preferred assessment formats during the COVID-19 pandemic, when traditional invigilated in-person assessments were not possible. Our main finding from the quantitative arm was that online OBEs resulted in the highest student satisfaction and were ranked as the option most students believed they would have preferred. Additionally, we found that in the case of assessment changes, an increase in weighting of future assessments was preferred over increases to weighting of past assessments. From the qualitative arm, we established that clear, timely communication regarding assessment changes, and consideration of student involvement in the decision-making process played a big role in increasing student satisfaction.

### Assessment format

- a. OBE authenticity

Online OBEs were the preferred assessment format reported in the quantitative arm of our study, despite CBEs being the most frequently implemented format. In the qualitative arm, participants also responded favourably to online OBEs.

Several previous studies have explored the usage and validity of online assessments, from both a faculty and student perspective. However, many of these were conducted prior to the COVID-19 pandemic where online assessment was a choice rather than a necessity. Durning et al. [17] conducted a systematic review of studies comparing OBEs and CBEs, and although they stated that the literature was limited, they had similar themes to our study. These included the perceived authenticity of OBEs, student anxieties around CBEs and issues about security and invigilation. More recently, Sam et al. [25] also demonstrated that students appreciate assessments being representative of clinical practice, shown by their approval of clinical prioritisation questions, and that they see OBEs as more authentic to clinical practice where information can be looked up [26–28]. In line with this, several students in our study suggested OBEs taught them skills applicable to real-life situations. Sarkar et al. [20] also made similar conclusions in their study on OBEs in the context of the COVID-19 pandemic, adding that OBEs encouraged students to engage in higher-order thinking and be more critical and analytical. However, this study also found that the majority of student respondents did not want OBEs, but this was attributed to logistical and technical issues [20].

- b. OBE and student anxiety

Studies from both before and during the COVID-19 pandemic also reported reduced student anxiety with online assessments and OBEs [17, 20, 29, 30]. Broyles et al. [29] specifically addressed the issue of tension and stress in their study, finding that 80% of the respondents felt less anxious and more comfortable with OBEs. Durning et al. [17] also explored student anxieties around CBEs, and their findings suggested that students could overestimate the impact that OBEs have on reducing their anxiety and enhancing their examination performance. They additionally found that students performed better in CBEs but acknowledged that this may be caused by unfamiliarity with OBEs, due to the very small uptake of this assessment format prior to 2016, when the review was conducted. Stowell & Bennett [30] found that students reported similar anxiety levels in online assessments and classroom assessments but found no difference in performance.

- c. OBEs versus CBEs – assessment driven learning

However, within our interviews students did report some drawbacks with OBEs such as the lack of motivation to revise for OBEs due to reliance on notes, leading to insufficient knowledge. Limaye et al. [28] and Sarkar et al. [20] also reported similar observations with the latter stating that students felt like they “copied from the book rather than thinking” during OBEs. Interestingly, some of the interviewees felt that questions in their online OBEs were harder and were phrased in a way that it made their notes less helpful than they would have hoped for. This was also a source of anxiety reported by students in the work by Broyles et al. [29]. The use of questions which are difficult to search online such as Clinical Prioritisation Questions or clinical case-based questions has also been explored in other studies and was found to mitigate concerns about OBEs being easier or requiring less knowledge [25, 31]. Furthermore, several studies raised the issue of online assessments and OBEs taking longer to complete compared to traditional in-person assessments as raised in our interviews [17, 20, 30, 31].

- d. OBE proctoring

Faculty [8, 32, 33] and students [26, 33] have previously reported concerns about cheating in online assessments by looking up reference material from notes/the internet in CBEs, and collusion with others, though there is a lack of consensus into whether online unsupervised assessments in fact do increase cheating [8, 34]. Previous studies have recommended online proctoring methods involving automated video systems to combat this, and our study also explored similar attempts made with supervised CBEs, showing that supervised CBEs were perceived as the second most ideal option after OBEs [31, 35, 36]. However, students in our interviews reported that they disliked online proctoring, and Cleland et al. propose that it is ineffective in preventing cheating [32]. Interestingly, Fuller et al. explored whether implementing online invigilation may indicate a lack of trust on behalf of the HEI in students and argues that we should trust in the morality and professionalism of future doctors to avoid breakdown of student–teacher relationships [8]. However, this contrasts with our findings where unsupervised assessments were ranked quite low by our students, with *n* = 36 students commenting in open text questions that they were concerned about other students cheating – “some students would cheat if it was unsupervised but closed book, whereas others would be guided by their moral compass”. However, one student expressed the belief that “as we are medical students…we aren’t going to cheat”.

The most commonly implemented assessment format in our study was online unsupervised CBEs, causing multiple students to report anxiety over peers cheating as discussed above. The importance of this concern is emphasised by the fact that perceived fairness was the most important factor for satisfaction within the student body.

OBEs, therefore, may be an effective alternative method to remove concern about cheating by looking at reference material, although they do not alleviate the worry of collusion raised by some students. However, a study looking at online open-book examinations in India during COVID-19 states that these students were significantly more likely to have inadequate time to answer questions, demonstrating that OBEs with an appropriate time frame to answer questions may reduce cheating [20]. Randomisation of question order for each student was also used in some cases to limit collusion [37].

### Assessment weighting

In our study, the lowest ranked assessment change was cancelled assessment with increased weighting on past assessments, even though this was the third most commonly implemented format, experienced by just over 20% of participants. Interview data shows this was partially due to an inability to be proactive and change past assessment results; as students felt like they were more in control of their grades and able to invest extra effort into a future assessment, even if added weighting to future assessments would be stressful. These results are supported by findings from Meccawy et al. which showed increasing past assessment weighting was the least popular option amongst students, due to similar reasons such as decreased opportunity to improve their grades [38].

Additionally, students felt anxious about not having the sufficient knowledge they needed to progress through their course if assessments were cancelled as it could lead to a lack of motivation to revise adequately. This was also expressed in our interviews by clinical year students about cancelled clinical skills assessments. Similar concerns about not learning the knowledge required sufficiently due to lack of motivation were also raised by students who had experienced formative assessments. Summative assessments with either pass/fail marking or with prizes were slightly more preferred. However, prior research suggests formative assessment can be useful in increasing intrinsic motivation and are good predictors of future summative assessment [39, 40].

Many students who experienced assessment cancellation or change to formative assessment also reported reduced anxiety; this was especially the case with those who underwent adverse personal circumstances. Formative assessments are commonly reported to reduce student stress [41, 42]. Others appeared to indicate that increased stress because of assessments during COVID-19 was preferable to the stress resulting from the reduced agency of using past assessments or the lack of validation from not having current knowledge being tested. Some HEIs made assessments optional during the pandemic, which could be a way of tailoring to individual student needs, allowing them to choose what they feel is best for them [43].

### Factors affecting preparation and performance

- a. Faculty communication and student involvement in decision-making

The COVID-19 pandemic has brought about many changes that could affect students’ assessment preparation. An important factor that impacted student preparation and satisfaction with the assessments was the communication from faculty about the changes. Many students expressed that clear and timely communication was key as it helped them better prepare and feel less uncertain for their upcoming assessments. This has been demonstrated to be especially important with the uncertainty surrounding COVID-19 [44, 45]. In general, a lack of regular communication from HEIs during COVID-19 was reported as a stressor by students in the qualitative component of our study, particularly when details about the assessment format were only released in the weeks prior to the assessment. The importance of higher education institutions (HEIs) communicating with students during the pandemic was analysed in detail by Al-Maskari et al. [46], who stated that “Communication with students is more crucial than ever. Therefore, HEIs should use all possible means to communicate necessary information to their students”. Fernandez & Shaw [47] also highlighted clear communication as an example of best-practice for academic leadership during the COVID-19 pandemic.

Whilst the absence of clear and timely communication was highlighted as a stressor by the students in our study, some students were consulted and involved within the decision-making process through a voting system. They reported being very satisfied with their assessment changes, as they felt they had more of an ability to influence whether the assessment format was the one they preferred. Studies on student involvement in decision-making have shown that it can increase transparency, decrease emotional distress, and in some rare cases, may even improve academic performance [48, 49]. Having a say in the decision-making process may have been empowering and have given students a sense of control in what were very uncertain and rapidly changing circumstances; this element could reassure students, even if the outcome did not result in their preferred assessment method. This was reflected by other published student opinions which urged future student involvement in decisions relating to unexpected changes in assessment [26, 50].

- b. Technical issues & familiarity with assessment environment and equipment

A wide range of personal circumstances affecting student preparation was described both in our interviews and previous literature [8, 26–28]. Some students described a positive impact on their academic performance due to a comfortable home environment during the revision and assessment taking period, and some students with physical disabilities found online assessments more accessible [32]. However, others described the negative impact on their academic performance due to: cancellation of clinical teaching; lack of access to usual equipment; technical difficulties; inability to focus in their home environment; physical and mental illness, and shielding under COVID-19 restrictions due to underlying health conditions. This led to an increase in contextual variability of the circumstances in which students participated in assessments.

A common concern amongst students within the literature and in our study was about the occurrence of technical problems, especially for online assessments [8, 31]. Technical issues affect both partaking in the assessment and the preparation in the run up to the assessment. Ilgaz & Adanir (2020) highlighted this in the qualitative component of their study stating “Participants experienced anxiety related to the potential for technical system problems, besides their own exam-related stress, in all online exams. Although none of the participants experienced technical problems, the possibility or potential for Internet connectivity problems, power-cuts or non-responsive computers was seen as a source of additional anxiety for the participants” [31]. Furthermore, a lack of access to adequate technology was also highlighted in our study, which has been found to affect some students to a larger extent than others [8]. Providing equal access to technical equipment and a standardised environment for students are important to ensure equity and fairness of assessment [8, 33], and to avoid exacerbating the already present social inequalities in medical education [51]. Further action to rectify this, as suggested by students in our study, could involve: providing a small number of participants with access to socially distanced quiet physical environments where possible, as well as HEIs providing technical hardware such as loaning laptops, and thorough pre- assessment checks of student and HEI equipment and software**.** Some online assessments during the pandemic ran asynchronously and could be accessed at any point over a 24-h period to prevent disruption due to lack of internet availability and to students sitting assessments in other time zones [52].

Several studies have found that students were concerned about a lack of practice and familiarity with the electronic assessment format [16]. This was also highlighted in the qualitative component of our study and our questionnaire found that familiarity with assessment format is the second most important factor contributing towards satisfaction. Both online and open-book examinations are new to many students and this lack of familiarity could impact process efficiency, study skills and preparation strategy. Some students suggested having mock assessments could assist with this, and the importance of mock formative assessments to drive student learning is well established in the literature [40, 53]. However, where mock assessments had been provided, students stated that the actual assessment had been much harder than the mock provided and therefore requested more representative mock assessments.

### Further research

Further studies are needed with equal sample sizes for the quantitative data collected on different assessment formats and weighting changes to increase validity and representativeness of the findings. Additionally, it is unclear whether these results are applicable to situations outside of the COVID-19 circumstance. For example, many students were very positive about their assessment changes due to an understanding of the challenging position medical educators were in. This may be demonstrated by the substantial use of modifiers –e.g., probably”, “perhaps”- used by participants during interview.

This research could be used for further studies to evaluate the importance of fairness, transparency, and authenticity to clinical practice in determining student satisfaction with future assessments. It may contribute to discussing the role of OBEs and remote assessments, both during COVID-19 and beyond.

Additionally, as per our findings, many students had not actually experienced the assessment format that they indicated was their preference and this could perhaps influence their choice with the “grass is greener on the other side” phenomenon affecting their satisfaction. Future studies could further look at whether having experienced an assessment format affected student preference, and could look at the relative ranking of all assessment formats instead of only looking at their top ranked assessment format. Since some of the students had already received their assessment results at the time of the study while others hadn’t, satisfaction with the assessment method may be influenced by this in some cases. To overcome this, opinions of students on assessment format should be assessed before and after receiving results; however previous studies did show no correlation between perception of academic achievement and actual academic achievement on online examinations for other university courses [32], though further research is required on other assessment formats and specifically for medical assessments. Studies could also explore whether prior or predicted academic achievement of students influences their satisfaction with assessment changes during COVID-19.

Longitudinal studies to assess any long-term implications of these assessments on students’ confidence, mental health, and career applications such as FPAS should also be undertaken [6, 32].

Further research could also be conducted into how OBEs could complement CBEs and whether to implement a combination of both in medical school assessments.

## Conclusion

Our study shows that out of the assessment formats experienced during COVID-19, students who experienced online OBEs (19.6% of total participants) were the most satisfied. Additionally, amongst all participants, online OBEs were stated as their most preferred ideal assessment option under the circumstances. Similar preferences have also been shown in previous studies [14, 25, 33]. This may have been because students found fairness to be the most important factor for satisfaction and online OBEs allow students to search their notes and the internet, removing one of the major methods that would constitute cheating in a CBE. Changing the assessment to an OBE also reduced student anxieties about assessment cancellations affecting future knowledge. It is important to students that HEIs communicate changes in a clear and timely manner and also try to ensure fair standardised test-taking by minimising technical issues. Efforts should also be made to provide familiarity with new formats through use of appropriate representative mock assessments.

These findings could guide medical educators about medical students’ views on assessments, when in-person assessments are not possible, and highlight in general factors that students wish to be implemented before and during the assessment period, to develop future assessments that align with student preferences and decrease anxiety.

## Supplementary Information

**Additional file 1**

**Additional file 2**

## Data Availability

All data generated or analysed during this study are included in this published article and the supplementary information files.

## Abbreviations

CBE: Closed-book examination
HEI: Higher education institution
OBE: Open-book examination
OSCE: Objective Structured Clinical Examination
PACES: Practical Assessment of Clinical Examination Skills
