# Supplementary material for: Medical students’ perception of changes in assessments implemented during the COVID-19 pandemic
Source: BMC Med Educ. 2022 Dec 7;22:844. doi: 10.1186/s12909-022-03787-9 (PMC9727955; doi:10.1186/s12909-022-03787-9)
Supplement: Supplementary file 1 — Additional file 1 [file 12909_2022_3787_MOESM1_ESM.pdf]

Medical student perception of changes in assessment style implemented during the COVID-19 pandemic

Interview transcript 1

Interviewer: Thank you so much for taking time out to participate in this interview. We really grateful. Before we start, I'd like to let you know that this interview is being recorded. Is that OK with you?
Participant : Yes no problem
Interviewer: Okay, great, I'd also like to add that your personal information such as your name or any uni names that you mentioned will be anonymized. When we finish this interview you will be sent a recording of it as well as the transcript. After you receive the transcript you will have two weeks to withdraw your interview or bits of the interview if you want to. You just have to email us if that's what you want to do so.
Participant: Okay perfect thank you
Interviewer: and feel free to stop me at point if you if you wanna ask questions, if you wanna take a break or if you wouldn't like to continue anymore. That's fine just let me know.
Participant: Okay
Interviewer: Okay, great, um do you have do you have any questions before we begin?
Participant: Nope
Interviewer: Okay, let's begin, then, um, can I ask you what were the changes to your exams?
Participant: so they move from having four separate full year exams to having one combined online exam, having our optional module exams cancelled and our OSCE exam has been moved. Initially it was to September, then it was to December and I believe now it's been moved to January. But there's still a misunderstanding of when it could fall.
Interviewer: Okay, so you had quite a lot of changes then, um, so you had four previous separate exams that is now a big one exam, did you say?
Participant: yeah
Interviewer: Okay, and how was this exam carried out?
Participant: It was an online open book exam using our usual online exam service provider. So we use robo for online exams, so it was still the same thing. However, it was an open book exam taken from your own homes or wherever you choose to be.
Interviewer: Okay, just to clarify this, for exams that you would have had previously, what was the format of that? If you had had it in normal circumstances
Participant: So we would have had a three MCQ exams. Multiple choice question exams and all online and there would have been one physical exam which would be an anatomy spotter exam as a part of it and the other part of it would have been an online prescribing and dosage exam.
Interviewer: Okay so all four would have been online.
Participant: Pretty much other than our anatomy supporter, which which would have counted for half an exam and would have been in person
Interviewer: Okay. So how do you feel about the spotter exam being online now?
Participant: So the spotter exam being online, I think it caused a few issues because of the fact that usually, when you are given a pro section to look at it's quite easy to do not too difficult, whereas using online imaging based on the quality of image or the quality of your screen. If the screen brightness is a bit low that could severely impact your ability to understand what the stuff youre looking at
Interviewer: Okay, that's very helpful to know. And can you tell me a bit more about this one big exam that you had so it was online and it was open book. How did you feel about being able to look at the notes?
Participant: I think um having access to notes was less beneficial than I would have thought so I think based on the content and the timing of the exam it was a lot more difficult to yeah. It's not the

same as being able to just sort of flick through for your notes and find the answer and the questions were worded in a manner, which or prompted you to think a lot more than would have been if it was usual in uni exams. And yeah, I think having the fact that it was open book, I think it didn't assist or aid in any of the ability to answer questions. So I don't think it's all made the questions any easier.
Interviewer: and how do you feel about that?
Participant: I'm I thought you know the University, they've done what they have to do with what they were given in the situation which they were in and and I think yeah, it's better that they sort of allowed it to be open book because I think that's a more understanding approach than universities or exams and courses, which sort of used eye tracking software and had people watch over you while you're taking your exams. I think yeah, this was a better system.
Interviewer: And you didn't have anyone supervising you?
Participant: yeah there was no supervision
Interviewer: Okay, So from what you said, did you mean that your questions had been changed slightly than what it would have been before so you couldn't look up information is that correct?
Participant: yeah, I think they just made it a bit more difficult rather than what would have had before. There was a lot more that was taken into answering each of the questions and understand the question in itself.
Interviewer: What are your thoughts on that change?
Participant: I think it would have been nice for them to make that clear. They did give us a mock exam but that mock exam was a lot easier than the real exam from everyone's perspective, so I believe that yeah, I think if they would have just highlighted that then maybe would have helped us prepare a little bit more.
Interviewer: okay, In terms of your optional exams you said there were cancelled?
Participant: Yeah, so we have optional modules, so we completed one prior to January last year and we had one which was due in multitudes away, so some of them would have been projects and some of them would have been coursework, some would have been written exams, some would be online exams and all of these sorts of assessments were cancelled.
Interviewer: These optional exams count towards anything in the year? did it count towards your final grade?
Participant: Oh, so no they didn't. Our main exam was weighted such that it would negate the loss of the exams we didn't do.
Interviewer: But before any of these changes, before Corona ,would it have counted, these optional exams?
Participant: yeah it would have counted to less than 4%
Interviewer: Okay and how do you feel about this cancellation?
Participant: Yeah, so I felt as if it was a good idea, and to not have the exams because we were unable to sort of complete the module fully so therefore, it was nice to not have to worry about that alongside your main exams as well as anything else that you're doing.
Interviewer: Okay and in the sense that your final one big exam counted towards more towards your final degree. How do you feel about that?
Participant: Oh. I think that. Because they did give us fair warning. I think it was up to myself what I wanted to do with it, and so I think it was because of the amount of time that we are given from when it was mentioned to ourselves and that exams would be online, and they did give us enough time to prepare so I don't have a problem with that.
Interviewer: Okay, I'm going to move on to the next question now, which is about your ideal exam type so normally if there was no Corona everything was normal, what would your ideal exam type be?
Participant: So, what do you mean by that?
Interviewer: So, things like would you have preferred it to be at hall would you have preferred it to be online? would you have preferred it to be supervised unsupervised?

Participant: Yeah, I think I'm quite happy to do online or even written exams as long the I don't have too many preferences. I think online has its merits. It's usually multiple-choice questions. If it comes to typing you don't have to type as much whereas with pen and paper ink may run out things like that hands may get tired.
Interviewer: OK, so you say you don't mind whether it's online or in hall. Is there anything else that would affect your ideal exam?
Participant: I think itd be access to revision material prior to exams or having knowledge of the layout or setup of the exam, and I think I'd be much happier being prepared even if it was one or two weeks in advance about whether it is an online exam or in person exam and if its long writing or multiple choice answer question. Just having a knowledge of the format of the exam, would help.
Interviewer: Um what about the sort of environment you're in does that matter to you, or not really?
Participant: I wouldn't say so, no. I think I've completed exams in noisy environments, quite environments. Ive completed exams outside inside. Haven't really seen too much of a difference
Interviewer: Okay so the main thing to you is being told the information before hand?
Participant: Yeah.
Interviewer: Now, what would you like, during the COVID-19 circumstances, for your ideal exam to be. What do you think University should have done?
Participant: I think I was quite happy with what they have done. I would have because a lot of us were in lockdown at home anyway, for me to then have to travel another two hours to come up for my exams, even if it was a single exam. For me to travel that extra time would have cause me excess stress. I thought it would just be sort of a discomfort more than anything. Of course, with people who had flight restrictions then it would have been impossible for them. So I think yeah, having it online at home was a good idea, and I think yeah, when it comes to supervision and things like that. I don't think it's necessary. I think at the end day if a student wants to go and look at their notes then you provide them with that open book format. If students want to interact with each other then that's up to them. If people want to do it at work, they can if they want to. I think down to students and what they believe is right for them.
Interviewer: Okay. thank you so much for telling me that. And in the sense of weighting what would you have preferred the University to do?
Participant: So for us the weighting for second year is 33% of our first bmedsci degree which happens after the end of 3rd year. I think I would have liked for them to reduce the weighting because the fact that rather than having four separate chances to do really well, you have now given individual one chance. If they're not as strong or something then they don't have the ability to make up for it somewhere else so I would have hoped for the overall waiting to go down. But that didn't happen and you cant do much about that
Interviewer: So you would have hoped for your second year exams to count towards a bit less. Is that what you're telling me?
Participant: Yeah?
Interviewer: is there anything else you'd like to add to that question about your ideal exam?
Participant: Nope
Interviewer: Okay, next question is a bit similar, which is what are the most important aspects to you about an exam for you to be satisfied with it. So what factors make an example good exam, to you.
Participant: I think, as I said, having a due time for preparation of the format of the exam, having due time to prepare for the content of the exam, knowing which exam I'm going to be sitting and then I think it's being given a fair amount of time to complete an exam. I think. If we were given unlimited amount of time for an example or an extra hour to complete an exam, I think it would be nice because it would allow for me to work at a slower pace if I needed to. However, of course, that may go and sort of increase the risk of cheating or increase whatever other issues the universities may have with doing that. I think just sort of giving a time frame where I don't feel rushed and I know

that's hard, cause that's different amongst everyone, but just giving that sort of time frame where Im not feeling rushed.
Interviewer: Okay, Other than the time frame and being given enough time to prepare, which is what you told me so far? Is there anything else you'd like to add?
Participant: no
Interviewer: So you mentioned that you would have liked extra time. Can you tell me a bit more about that?
Participant: Yeah, so like towards the end of the exam, pre-covid, like you'd finish with a few extra minutes to spare or sort of like just bang on time. Whereas with this exam, I felt very rushed towards the end, where it got to a point where I had five questions left and I had it to do that within the next 2 minutes, so for a fact I knew was quite rushed and even before then I felt the time pressure a little bit and I think that would have definitely impacted my ability to answer the questions as accurate as I could.
Interviewer: can you expand a bit more and why you felt that you had less time now than you did before?
Participant: See I'm not sure I think there may be a factor in terms of we were given more questions within a similar period of time, but that may not be the case. I believe the uni probably would have sort of organized the timing properly. I think it was more about the fact that because it was this open book exam, I did believe I had enough time to flip through my notes or first, probably, half of the paper but then I realize actually by the time I'm going through my notes and checking anything. It's easier for me to just sort of go with whichever answer I think is best and so I think it was my lack of these open book exams or lack of practise of these open book exams that made me feel like I didn't have as much time as I may have had.
Interviewer: In an ideal environment, how would you have liked to change that? You mentioned having a bit longer period of time? Is there anything else you would have liked done?
Participant: No, I think just having a bit more time would have been nice.
Interviewer: Okay, then let's move on to the next question compared to before, before covid-19 period, and now how has your confidence changed going into the exam? So did you feel more confident? less confident? it didn't change at all?
Participant: I think I felt slightly less confident going into these exams because of the fact that with previous exams I'd had that experience, I'd had a lot of practice questions before exam even started because we have practice exams every six weeks, whereas with the covid test we only had one mock exam and whilst sitting that mock exam I was at work, so for me it was quite difficult and I think a lot of that is personal issues. I probably shouldn't have been working while sitting my exams. But yeah, just in general, there was a bit less practice then what I would've hoped for.
Interviewer: Is there anything else other than practice that affected your confidence going in?
Participant: No I wouldn't say so
Interviewer: Okay so nothing about the change in format or weighting or the environment.
Participant: No, actually I would say that none of the environment or anything like that. I think it would be more of just teaching itself, I think because teacher moved to online teaching. So for the last three or four weeks, I felt as if I didn't learn as much as I probably should have in terms of content for my exam. And so I think that sort of didn't have a big weighting because a lot of the final weeks didn't have too much content in them anyway. But I think it was more of a mind game for myself. I think it was more of a psychological issue about not feeling prepared even though there wasn't that much content which I missed out on due to going home. It was more about just feeling as if I hadn't learnt that content.
Interviewer: okay, thank you. That's really helpful to know. Was there any other circumstances leading up to the exam that affected your confidence?
Participant: No, I don't think so
Interviewer: okay that's all the questions that I have do you have anything else you want to add that I haven't asked about? Anything else you would like us to know?

Participant: No
Interviewer: Okay thank you so much for taking your time out again. This has been really helpful for us. Ill make sure that you get the transcript and the recording after this interview. It might take a while for you to get the transcript, but you should get the recording as soon as we're done.
Participant: Okay no problem
Interviewer: Again, thank you so much. It's been really helpful.
Participant: No worries, best of luck with the study
Interviewer: Thank you. Bye.

Interview transcript 2

Interviewer: Also, just like to add that your personal information such as your name or any uni names that you mentioned will all be anonymized. When we finish this interview, you'll be sent to recording of it via teams and then soon after you should also get a transcript of it. Once you receive the transcript, you'll have two weeks to withdraw your interview altogether or any bits of the interview that you would like. You can email us what you would want us to change. And feel free to stop me at any point during the interview if you if you want to no longer continue, or if you just want to take a break or if you have any questions or anything like that.
Participant: Yeah, that's fine.
Interviewer: Okay, so before we begin, do you have any questions?
Participant: No, not really
Interviewer: Okay so I'll start then, so what were the changes to your exam?
Participant: So you know it was my third year when this like all happened so like basically what happened was we usually have an integrated summative exam at the end of the year which is an OSCE and a written paper. But both got cancelled. The OSCE just didn't happen and we didn't have any like clinical assessments. But the summative exam changed to a formative paper, and it was open for two days online. So it didn't really count towards our like final grade or anything.
Interviewer: Okay so for this study we will mostly be talking about the written exams. Was it just the one written exam that you had?
Participant: yeah, so we have two papers on the same day so it's basically just one exam but yeah.
Interviewer: Was that how it was meant to be before Corona as well?
Participant: yeah, so like before Corona. It's usually two exams on one day after Corona it was just like one paper, which was shortened so we had half the number of questions and it was open for two days online.
Interviewer: Okay can you tell me a bit more in detail how you carried out this exam?
Participant: Yeah, so um we got told about this pretty early on, and then like when so it opened two days before the original exam date before Corona so it was around the same period and it was on top hat I think yeah, top hat. And it basically the same stuff like the questions with the same structure but they were there was just less per module because our exams are like integrated so all the modules are in like 1 paper and they are weighted according to like the weightage of like the modules itself and then. Yeah, we just had like we just selected answers online and then after the exam we were able to check the answers according to what the examiners had put in like was right or wrong and we were allowed to like access the paper as many times as we like and they didn't really like, they said like the time was recorded like how much time you're on in Like the web page, but it didn't count for anything.
Interviewer: Okay And throughout this whole time, was it at all supervised or not.
Participant: Um, no, not really. So we had like a contract or like a statement or something saying like the normal like assessment criteria like don't work with anyone else. Use this like as an opportunity to test your knowledge and with all the general rules like I mentioned like it's open for two days like so and so on and so on. It was open-book so we could use any of our notes. Yeah, but other than that there wasn't really any monitoring.
Interviewer: Okay so first of all let's start with it being changed to a formative? How did you feel about this?
Participant: Initially I was quite happy because like obviously there's less stress then because its formative. And um because, like, at the time we were on placement like full days like no one had really started like revision and for us in third it's very much like self-directed learning so it's quite hard to learn the content whilst you're working placement like full days like you probably understand. So like I feel like not just me, but like everyone is quite behind on like that revision so we were happy initially that like it got changed to formative but then like given like a month or two into locked down and we actually started to realize like the implications of it being changed to a

formative and that's like I think when everyone started to get bit worried. Because it's a formative we can't like it can't count towards like F pass applications or ranking and a lot of people were banking on these exams to like maybe like improve their grade or Just improve their ranking in general because our third year exam usually- they're not considered easier, but because there are a lot more clinical side of things people tend to do better in them then second year because our second year was really difficult. Yeah so I think like for me at least it was quite- It was a bit like disappointing and like I didn't understand the consequences at the time but looking back now it's just a bit annoying that you like lose an opportunity to improve your grade or you just lose an opportunity to do anything.

Interviewer: Yeah, I can understand. And what about your feelings on it being changed to an online platform?

Participant: Um online like I didn't mind as much because, like, obviously it didn't count towards anything. If it did then i think that would be that worry like how they gonna monitor cheating and stuff, but because it was formative, and it was open for two days like time difference- because a few of my friends are international I would have been worried for them if like it was only open for like 2 hours and they had such a big time difference. So I think like yeah, it didn't really like make much of a difference to me.

Interviewer: Okay yeah you mentioned some points already about it being open for two days do you have anything more to say about that?

Participant: No not really.

Interviewer: Did you feel like it helped you more or it didn't really help you at all or-?

Participant: Um, I appreciate being open for two days because then that allowed me to choose time suitable to for me because, like often like at that I'm still like online schooling was still happening so it would have been a clash like with Wi-Fi and stuff. It was just a big issue like if you have like other people in the house like working with the same Wi-Fi it would have been very stressful if it was not open for like a prolonged period of time because like what if the Wi-Fi cut out and then the system crashed and all of that so that would have been really stressful. So, like in terms of it being open for two days, I think it was the most appropriate action of the University I feel

Interviewer: Okay. Thank you for that information. That was really helpful. Um so in terms of your exam weightings now? How do you feel about the changes that have been made?

Participant: I'm actually not entirely sure how they have changed because I'm integrating right now, and I can't remember if they told us. Actually, no they did. They sent us an official like document statement kind of thing saying how it's going to change so they've said that our 4th and 5th year are going to matter more, but obviously because of F pass application, they happen before your 5th year exams. So, our 4th year will essentially matter more. And they're not going to change the weighted of the 1st and 2nd year, which personally I find that I think that's fair. Like with everything going on obviously it's not ideal, but I would rather have future exams having more influence than previous exams because I can't change my previous results. So, if they were good if they were bad like I can't do anything to change them. But at least like even though it will be more stressful when I go into 4th next year as I have to perform better but at least I have the chance. And I'm like aware from beforehand like okay I'm going to have to put work or I'm going to have to focus on this, so it gives me an opportunity to actually take initiative. But if it was the other way around and they just like increased like the importance for older years I don't think I would have liked it as much.

Interviewer: Okay, so from what you're telling me you have quite a positive outlook on this increased waiting on future exams.

Participant: Um kind of like I'm trying to be as positive as I can because, like for our uni our 4th is really, really challenging so it's not gonna be easy. But I'm like I think it's the best-case scenario, I've thought about that quite a bit. I would much rather like I can do something about it then like not being able to control what's happened like in the past, so I’m not entirely positive, but I'm optimistic

Interviewer: that's good. Okay let's move on to the next question then. What would your ideal exam type would have been like had it not been for Corona so in an ideal world where everything was back to normal. What would you say your ideal exam type was?
Participant: So, I'm not a big fan of exams in general, but like I know that they have to be done, so I think I would have like. I think I would have liked the way it was going to be, so like having two papers, one in the morning, one in the afternoon. Like Yes, it would be a long day, but that's the way it's been in my first and second year, so I will have like I would have preferred it just to remain that way and then having like the clinicals like the way they organize on different day. Because it just Like I feel like that would just be the ideal situation rather than splitting it across like multiple days as that would have been stressful.
Interviewer: Okay So what are what are your reasons for preferring to sit exams in an exam hall all done in one day?
Participant: I think it's more because, like it's just one day that I hope to work for and then after that, like I can enjoy my life again. But like but if it was like dragged out like a levels and GCSE's over like weeks. It's just a much longer process and honestly like it's just too much stress so yeah. And it just gives me a bit more focus. Like if it's on one day then I know like Okay this is it like I have to get it done by then rather than like in A-levels when you split it across lots of days. Then you like focus your revision for one thing and then you have to be tactical like what you're revising when. This is just like everything is due for this day. Yes, it's stressful. But like you just have to do it.
Interviewer: And what about um in terms of format or the environment like what would your ideal format be.
Participant: Like format in terms of like the style of questions or format in terms of like the paper?
Interviewer: So, format in terms of it being in hall, or online or being supervised or unsupervised that.
Participant: I think like in an ideal world I wouldn't mind it like supervised in an exam hall. It's just what I'm used to and its not been an issue for me in the past, it's just normal exam conditions. It's just kind of what's expected, so ideally, I would prefer doing like in person in an exam hall.
Interviewer: Okay, what about um preference in terms of format in the sense of It being a paper or a coursework or an essay or some other kind of assessment.
Participant: So, I don't like essays at all, they're not my strong point. So I definitely would prefer like a written- like an MCQ paper. So, I've been having MCQ paper since like first year so I've never had to write an answer in my medical school exams. So it was like it's hard at first, but I would prefer an MCQ paper the way I've had experience in. So like clinical cases and then like you get like 4 questions on the same case or something like that just because I'm used to it now like.
Interviewer: Yeah, that's totally understandable. What about now that we've been affected by COVID-19? How would you have wished for your exams to be carried out? Since we can no longer do it in halls.
Participant: So I like obviously liked it being formative. But if I wanted it to be summative then I would think of maybe like an online paper. Which I think it should be open for like maybe like 2 days. But then I understand that if it's open for such a long time, then. It can't not be open book, it would have to be open book. Making the best out of like the situation we are in that's probably why I would prefer.
Interviewer: Okay, thank you so much for that information. And in terms of factors that are important to you, that make a good exam, what would you say are the factors that are most important to you. I know it's a bit repetitive
Participant: No, it's a different question. It's not repetitive. I think for me to assess my knowledge, especially to do with clinical stuff and application as I started my clinical years, I would prefer like a clinical case and then ask questions from there because it's more like what we would be experiencing in real life so it's more like applicable than like people just being asked to recall like really like random facts about like a certain enzyme like I think its fine in first and second year as that's what we've been learning fine like ask us that, but like I prefer like having a case to work from.

Interviewer: Okay, what about in terms of things like carrying out the exam itself, so maybe things like access to the exam so how easy it is to access the exam, how easy is to access Wi-Fi maybe, are things like time zone like any of these things you would hold as being important to you to make it a good exam?
Participant: I think like time zones do matter but obviously I live in the UK so it doesn't like affect me as much, but because, like some of my close friends do you live in abroad like it does affect me in that way, like I care about them I would want them to be able to do the exam properly, but even though it doesn't like effect like my paper. But Wi-Fi Wise like Yeah, like I think in Quarantine everyone had like difficulty with Wi-Fi, so in that circumstance I would not like the pressure of having like Okay your papers open for two hours. You have to finish all between that and then like it would just make me a lot more like worried just in case like the Wi-Fi crashed. Even if it didn't like I would just be preoccupied thinking about that. And then like I didn't mind like sitting at home because, like my family were understanding and I was comfortable in my own room and I was able to like sit the paper and I had like snacks and food and water like I was basically like in my ideal situation like even though it wasn't in the exam hall.
Interviewer: Yeah. That's fair enough. And finally, I have one more question which is compared to before the COVID 19 pandemic and the changes that have occurred. How has your confidence level changed going into the exam? Or maybe it stayed the same.
Participant: Confidence level regarding like my knowledge or confidence level just like in general?
Interviewer: Just how confident you felt going into exam due to several reasons it could be.
Participant: Okay, I wasn't confident at all going into like my paper because- because it was formative I didn't really worry about my confidence going into the paper. So, I don't really think about as much, but otherwise like I'm quite anxious when it comes to exams. So I didn't have that, like that anxiety related to the exam, but at the same time, I didn't learn anything for this exam like I didn't study properly because I had already known it was a formative, whereas if it was a summative I probably like the time they released the statement and the time, like we all went into lockdown would've probably been the time I would have started like properly working and like pushing myself for the exam so in terms of knowledge I wouldn't say that I would have been confident to go into the Summative. But I didn't really care for the formative
Interviewer: From what you're telling me, a major factor in this seems to be that it was formative. Is that correct?
Participant: Yeah, I think the whole perspective would change if it was like actually counted towards my degree.
Interviewer: Okay. Was there anything else any other circumstances leading up to the exam that affected your confidence?
Participant: No, not I think just the way like I am as a person like my personality like when it gets to exam time I get nervous a lot but that's just normal yeah.
Interviewer: So, there's nothing else that you want to comment on in terms of your confidence?
Participant: Not really, like if you have any other questions I'm happy to answer them.
Interviewer: Um no actually that's the last question, we had. Do you have anything else you want to add that we haven't asked about maybe?
Participant: Not really, but if you guys need like me to answer anything like in the future or anything like you forgot to ask, then I'm more than happy to do that.
Interviewer: Thank you so much. No this has been really helpful. It's been really good. Thank you so much for your time. Yeah, you should be expecting the recording very soon and the transcript, hopefully in the next few days.
Participant: Okay
Interviewer: feel free to contact us, if you'd like, about anything
Participant: Okay thanks so much
Interviewer: Thank you bye
Participant: bye.

Interview transcript 3

Interviewer: And yeah, I just wanted to remind you that everything, all of your personal information, such as your name or any uni names that you mention, will all be anonymized and when we finish this interview, you will get a recording of this interview as soon as it finishes, and then you'll also get a transcript a couple of days later. And when you receive the transcript, you'll have about 2 weeks to withdraw your interview or bits of the interview. And to do this, you just need to send us an email just telling us what you want to do with it.
Participant: Sure.
Interviewer: If you don't want to do anything, then you don't have to tell us anything.
Participant: Sure, thank you.
Interviewer: And yeah, feel free to stop me at any point if you don't want to continue the interview or if you want to take a break or if you just want to ask questions or anything like that.
Participant: Yeah
Interviewer: Feel free.
Interviewer: Yeah, so before we start, do you have any questions?
Participant: I'm good.
Interviewer: Ok, let's start then. So what were the changes to your exam?
Participant: So I'm in second year.
Interviewer: Ok
Participant: So what they did was they, when everything, when the transmissions in the UK is gone up, they said that we could still have exams, they hadn't cancelled it at the beginning, so they were thinking of moving it online. Then a week or two later- this is around March, so we haven't really finished our second semester yet. And then around- I think around the start of April, they sent an email saying that all exams for the year were cancelled.
Interviewer: Ok.
Participant: that we would do what they call bridging coursework instead, in place of exams, For those coursework- the written coursework were basically assignments that they made, that they created for us to do. And then we- we do them, and then we submit them onto Canvas for them to mark. So what we had to do was create a poster in anything that we are interested in, that we kind of covered in the year, but not really.
So kind of like an extension of our learning, so we had to make a poster and for everything that. We also did Canvas quizzes and we use like Clinicalkey, I think there's a thing called Clinical Key- there's quizzes on there that we had to complete.
Interviewer: Ok
Participant: And send it off to kind of like let Med school know that we've done them.
Interviewer: Ok. And ok, so all of your exams were cancelled, how many exams were you meant to have before?
Participant: So how it works at X is that we get one exam at the end of the Christmas holiday, so January we do a MCQ paper then and that's for Sem 1. And then the rest of the exams will be covered in the May exam period. So that would be the second half of the Sem 1 paper. So we did- we did MCQS - multiple choice- in January so the second part would be short answer questions. So we would have to do that to complete Sem and then we would do an MCQ paper and an SAQ paper for Sem 2, all the things we've done in Sem 2. And then we would do a regional anatomy paper, which is basically an essay. But you have to like do it in the exam hall. And then there's this thing- we have this module called PAS- so professional and academic skills, that's what it stands for – that, that we had to do at the end of the year in the May period as well. And I think, for second year, we would have done an OSCE exam and a Prosec exam as well on top of that.
Interviewer: And all of those were cancelled?
Participant: Yeah, they were all cancelled.

Interviewer: Ok. So the coursework that you mentioned - how was that carried out? Did you have to- How did you carry out the coursework?
Participant: So-
Interviewer: As in, was everything in person or online or, stuff like that?
Participant: It was all online. They sent us emails on where we can find resources and where we can submit our, our work and stuff for the coursework. It was all on- so we submit it all on Canvas. So yeah, so it was all online. There was nothing really in person.
Interviewer: Ok, and what were the timings of this coursework?
Participant: They- so they kind of set the information out around the start of April.
Interviewer: And they originally wanted it all to be done before the start of the new year, so they wanted it to be done like June or July. But then everyone in the year complained about how they're- there might not be enough time for them to do it because quite a lot of people helped out during, during like when the lockdown started, in hospitals and stuff so they wouldn't have time to do all the work. So the Med school kind of said as long as you do it before September for most of the work then that should be fine. So that was kind of the deadline.
Interviewer: Ok. And, how do you feel about your exams being cancelled
Participant: I feel- I don't know- like that it's good that they're cancelled because it's like a weight lifted off your shoulders, but then at the same time it's kind of frustrating, because. I didn't really prepare probably as hard as other people have started to prepare for exams, but I can imagine if you've been studying for a while and then it's been cancelled that would be quite annoying.
Interviewer: Yeah, I can imagine.
Participant: There's just so many uncertainties of how they're going to weigh up the deciles, things like that, which Med schools still haven't really gave us quite a lot of information on so I don't really know what's going to happen with that, but the fact that they made us do the bridging coursework was- I quite liked it- because then it's not just like everything I've learned in the year. I kind of just "Oh, I've learned it. I can't really do anything with it". The bridging coursework kind of made me do something with it. So had to do a bit of work, which was quite nice.
Interviewer: Yeah, and this coursework - was it assessed, like were you assessed on it?
Participant: Some of it were formative, so the Clinicalkey quizzes they were just for our own kind of understanding and consolidation, but some of it were marked. So the poster we had to submit them to be assessed, and some of the Convos quizzes as well, they would assess those too.
Interviewer: And were these, so they were summative and did they count towards your year grade? Or towards the final degree?
Participant: I think, as far as I know, you have to do them- you have to pass them in order to progress to the next year. In terms of… if they give you an actual grade, or if it affects your deciles, at the moment X- they sent out an email the other day saying how they're still kind of working on it. They've got a few options for that. I don't know what the options are. They haven't said, but they said that once they kind of discussed it and are happy with like the two or three options, they're going to release the options and make everyone vote on it, and then whichever option gets the most amount of votes would be the one that they would use to weigh up their deciles and see what happens.
Interviewer: Ok, and how do you feel about this whole process?
Participant It's been like- I think they- everyone's a bit worried when they canceled exams, but then even now they still kind of haven't really given us an answer. So I'm just a bit kind of just floating around. I'm not frustrated because obviously for me it's- I don't really think about it too much I guess. But yeah, it's just a bit annoying how I still don't really know what's going to happen.
Interviewer: Yeah I can imagine.
Participant: Or I don't know if I should like work really hard for my next exams because I don't know if they will count more. Yeah, so I'm a bit annoyed. But at the same time, I'm glad that they're doing like a kind of mass voting system, so it's not like we- like as a – as a cohort we kind of can decide what happens as opposed to just Med school deciding for us.

Interviewer: Yeah, and currently you don’t really know the options, do you?
Participant: No, they haven't really discussed options at all. They just said that they have options, they didn't really say what they were.
Interviewer: Ok. Ok, so yeah, your exams were cancelled and you had coursework. And what about in terms of weighting, do you know how things have changed?
Participant: Yeah, it's the same answer as before I have no idea. Med school said that they're working on it, but they haven't really said what they're going to do about it.
Interviewer: Yeah, so you don't know if previous years will count towards more or if future years will count towards more.
Participant: Not at the moment.
Interviewer: Or if it won't change at all, ok.
Participant: Yeah, not at the moment, I don't know at all.
Interviewer: Yeah, so I'm guessing you feel similarly towards that as well.
Participant: Yeah it’s all up in the air a bit.
Interviewer: Yeah
Participant: Yeah
Interviewer: Ok and have they told you when you will find out- you said soon, right?
Participant: The email- I'll see if I can get the email up. I don't know if they gave us like a time scale but I'll double check.
Interviewer: No, don't worry about it. It's fine, don't worry about it. Alright, OK? Let's move on to the next question, which is what your ideal exam would have been like. So what would you have preferred to happen?
Participant: I think I would have preferred to have exams anyway, just because then the whole weighing thing and everything like that would be less confusing and it would be a good way for me to kind of, you like, kind of consolidate everything I’ve learnt and actually use- use my knowledge, so I think I would have preferred an exam, but. I probably would have- I don't think I would have been comfortable with like a sitting in exam, so probably an online exam. I think that's probably what I would have wanted.
Interviewer: Yeah, can you expand more on why you would prefer an online exam?
Participant: Just because I think that online exams most people will be able to like sit,sit it and be able to, do that, do their exams like even if you're international, if you're not really based in your uni at the moment, maybe you've gone back home. It's just like a good way for everyone to access. I understand that there could be a lot of time differences and things and like really bad wifi on your laptop and things like that that may prevent you from doing it, but I think the majority of the people will be able to do online exams. I think that's why I would prefer that.
Interviewer: And do you have any preference in terms of these exams being supervised, unsupervised, open book- anything like that?
Participant: I think even - even if it is supervised or unsupervised and open book, there will be quite a few people that would- well not cheat, but like there will be a few people that would act as if it's open book anyway. So I think if they - I would prefer it if it's unsupervised, just because I think if it is supervised then it'll get a bit complicated in terms of how it would work. Whereas supervised, I imagine it would just be like on the system you answer it in like an online format, which will be a lot more easier. So I think I would prefer it to be unsupervised. Open book or not open, I don't think it makes a massive difference because some people would treat it as an open book anyway. So…
Interviewer: Yeah.
Participant: Yeah, I mean I would probably prefer open book just so that I can get a better grade on my exam.
Interviewer: Yeah, and I- I'm not sure if you mentioned it, but in terms of the exam being either formative or summative, which one would you have preferred?
Participant: I think if the exam was how I would like it to be, which would be online open book, then I probably would quite like it to be a summative exam.

Interviewer: And why is that?
Participant: Just so that it counts for something?
Interviewer: Yeah
Participant: And if it's online and being open book, obviously I probably will do quite well. So then I would quite like it to be a summative just so that it counts. Just in case I don't do as well later on down the years.
Interviewer: Yeah ok, and in terms of if we were still in the pre-Covid situation and nothing has- nothing's changed. What would you have liked? How would you have liked your exams to be carried out?
Participant: If nothing has changed, then I wouldn't mind it being like a sit-in in person exam or an online exam, because an in person exam will be how things normally would be, so I'll be used to that, then I wouldn't mind doing that. But then online I wouldn't mind either cause I'll probably do better.
Interviewer: OK, thank you so much for all of that information. Let's move on to the next question, I think, which is a bit similar, but- For you, what are the most important aspects of an exam that make it a good exam and when we ask this question, we're not talking about the contents that's in the exam, but more the exam itself.
Participant: I think the important- like when they test you on what you actually have learned during the year and what you- what they think is applicable knowledge for like you're like when you go on- when you go on and do clinical medicine and go on placement and stuff, I think that's why exams are important. I don't really like it when they ask you like specific questions on specific diseases or specific medication that you like just memorise but you don't actually- you might not actually use that information later on.
Interviewer: And, what about things other than the content of the exam? So how the exam is carried out, where it is carried out? Timings, you know, accessibility to the exam?
Participant: Yeah
Interviewer: What about those things? Which things would you consider to be the most important?
Participant: I think timing- timing of the exam and things like that. And preparing for the exams will be quite important. Preparing being like revising for it. So obviously you can find out if you can work efficiently or not and this whole thing of the exam will be putting you kind of under pressure to see if you can work well under pressure, and also if you can manage your time well during that period of time. So I think those are quite important skills. In terms of accessibility, like to actually go to the external sit down. I don't think it's that important. It was- I think it's important in terms that you're - like you turn, you turn on-you turn up on time- like punctuality but you can do that when you're online as well.
Interviewer: Yeah
Participant: You don't have to, you know, travel and show up to show that you can do that.
Interviewer: Yeah. Any other points?
Participant: What, what was the question? You mentioned quite a few points, I don't
Interviewer: Oh sorry. So other things that you could consider could be, fairness of the exam or like time zone wise, you know if you are international for you it might be know if I managed-important that the exam is done at a certain time, because that might affect you. Just things like that.
Participant: Yeah, then in that case I think yeah, so the exams were online then it would be quite unfair to a lot of people that are international students just because of the time zone difference. Because obviously there could be a big time difference and then they would have to change their schedule which would be unfair to them. But also if it's online, like you said, it can't be supervised like an in person exam, so a lot of people might look through their notes or Google things then in which will be unfair to the people that have taken it as if they were doing it in exam condition and have done it just using everything they've learned.
Interviewer: Yeah
Participant: And I think it would be - online exams, as well would be unfair for people that might not have the best computer or the best Internet connection. Or if they are living at home, their

environment at home might not be the best for them to study and things like that. So I think an in person exam where you sit down it's fair for those things because everyone is kind of in the same condition, doing it at the same time and being all supervised. There's no kind of variable in that.
Interviewer: Yeah. Yeah, there's definitely positives and negatives for both of them. Yeah, thank you so much for that. OK, let's move on to the next question then, which is compared to how your confidence level would have been like pre COVID-19 era, how confident were you going into this exam? So I guess your exam was cancelled, so maybe the coursework, how confident were you doing the coursework?
Participant: I was… quite confident, the emails were explained quite well and if you if you didn't understand them, you could ask them questions, they replied quite quickly. I did find it a bit difficult in terms of finding - finding free time to do them because they were quite a lot and they all had varying deadlines.
Interviewer: Oh ok.
Participant: And obviously loads of different people mark them so it was not - if it was a normal pre-Covid kind of schedule then it wouldn't be quite a lot of work, but because it was during Covid and I was one of those people that were basically working full-time in hospitals helping out-
Interviewer: Oh ok.
Participant: I found it quite challenging trying to manage my time and having to go to work but also having to do work and balance.
Interviewer: Yeah, I can imagine that, yeah
Participant: Yeah
Interviewer: So I guess the circumstances that you were in did affect your confidence a bit.
Participant: Yeah
Interviewer: Ok
Participant: Not in terms of how to do them, but in terms of getting it done in time, yeah.
Interviewer: Oh ok and how… did your confidence change in terms of your exams being cancelled?
Participant: It was quite a - It was quite a relief for me getting exams cancelled because there were, like I said earlier, that only one paper was sat in January and obviously the rest would be sat in May- that would be quite a lot of- and term would finish end of March so that that would realistically give me five to six weeks to try and learn a whole year's- try and learn and revise for a whole year's content so it would be very stressful having to do all of that in a month and a bit.
Interviewer: Yeah definitely.
Participant: So yeah. What was the question again, sorry?
Interviewer: It was just how confident you felt due to the cancellation of your exam. Whether your confidence increased or decreased.
Participant: My confidence…Well, like I said, I felt really relieved that they were cancelled because then I didn't have to stress myself out for a week and a half trying to learn everything. But going into third year, I probably would say that my confidence isn't as great because- because I haven't been tested on the knowledge, I don't know if I've learned everything.
Interviewer: Yeah
Participant: In order for me to be able to go on placement in third year and having to like answer all the questions the consultants ask me, if that makes sense.
Interviewer: Yeah, that's definitely understandable, yeah
Participant: Yeah, because there wasn't like a time pressure for me to learn everything. I kind of just spaced it out and learnt some things and haven't really gone over some other things. Whereas if I had exams I would have had to learn them whether or not I liked to.
Interviewer: Yeah yes, that's a very interesting point you make. You did mention that you were working leading up to the exam. Was there anything else leading up to the exam that affected your learning or your confidence? Or anything like that?
Participant: Yeah, so I was working, but also the fact that I went home before lockdown was announced, the start of lockdown was announced I think the 23rd of March.

Interviewer: Yeah
Participant: So I- because I was scared that if exams were to carry on, I don't do very well revising at home. I live with my sister and we're very close so she disturbs me all the time. So that's a big distracting factor.
Interviewer: Yeah
Participant: So I, when lockdown was being announced, because the exams weren't cancelled at that point because they were cancelled in April, I was kind of like right, I would prefer to revise in X in my student house rather than at home. So I moved back to X.
Interviewer: Ok
Participant: The day before lock down just so that if exams were to happen, I wouldn't, I would be in the best situation to study for it, but then moving back I was one of the only people that kind of did that, so I did feel very lonely living in a house-
Interviewer: Oh no, yeah
Participant: And also working and you know, being busy and being stressed so that, that I didn't really enjoy that, being by myself.
Interviewer: Yeah, I'm sorry to hear that
Participant: That's alright. No.
Interviewer: Yeah, that that must have been a quite stressful period for you then.
Participant: Yeah
Interviewer: Any other points that you want to make?
Participant: Um, not for that question, yeah, I've yeah
Interviewer: So we've talked about the circumstances, and what about your confidence level in terms of weighting? So all the weighting changes that's been happening or that you don't know of? How-How's your confidence level been affected due to that?
Participant: I think it definitely caused more stress for me the the start of this academic year because regardless of whether that we had exams or not, because, actually yeah like because the exams were cancelled they're either going to probably use this year or the year before. So first year, yeah, they're going to weigh those more. And I know my first exams I did OK, but I didn't do amazing, so in my head that means that I have to do really well this year to try and average out my decile in my weighting. So I kind of stressed myself out a little bit and I kind of put a lot more pressure on myself to be on top of everything this year, just in case.
Interviewer: Yeah
Participant: -it does- they do like kind of change it so that this year weighs more, then at least I'm prepared for that. So my confidence is probably below average.
Interviewer: Yeah, I can imagine, it's quite uncertain times.
Participant: Yeah, exactly.
Interviewer: Yeah. Yeah, that's all the questions I had for you. Thank you so much for answering them all so well. Do you have any other comments that you want to add? That I haven't asked about or anything.
Participant: I just think, not really. I think it's just more to do with online learning and how it's very different to kind of-
Interviewer: Yeah, definitely
Participant:- in person exams, it's all a bit strange. I don't think that a lot of medical schools really know what's going to happen and how they're going to- they're still adapting, so I think that's one of the uncertainties.
Interviewer: Definitely, yeah
Participant: Things can change at any minute all the time.
Interviewer: Yeah, I think we will have to continue with this online learning for quite a while.
Participant: Yeah, yeah exactly. It's all very unknown.
Interviewer: Yeah, that's- that's for sure, yeah. But yeah, thank you so much for taking time out to do this interview. It's been really, really helpful for us.

Participant: That's ok.
Interviewer: Yeah, after we've finished you will get the recording- as soon as we're finished as I said before and you should get the transcript in a couple of days. Yeah, thank you so much again for your time.
Participant: Can I also- can I ask one more question, sorry?
Interviewer: Yeah sure, sure
Participant: Just came into my head. So this research that you're doing, do you know what you're going to? Are you going to publish a paper? What are you going to be doing- do you know?
Interviewer: Right now we're still conducting the interviews and getting the data from it, but once we have done that and we have analysed everything we hope to publish this and hopefully make universities as well as, um, you know, education boards more aware of student choices, which will hopefully have an impact in future exams.
Participant: Sure
Interviewer: In a good way, hopefully
Participant: Do you know if it becomes published or like if you've finished writing it, if I could read it and stuff, would you like-would you be able to update people that have taken part and stuff. Do you know that's possible?
Interviewer: I mean, honestly, we haven't really discussed that yet, but I don't imagine why not- why we wouldn't be able to share the paper with – the paper with you guys. So yeah, definitely. if that's something you're interested in, then –
Participant: I would be interested in seeing what you find out.
Interviewer: Yeah
Participant: I think that'll be really-
Interviewer: Yeah definitely. We will keep you updated.
Participant: [laughs] Thank you!
Interviewer: Yeah, we hope this will get published. If you don't hear from us, that means it hasn't been. But yeah, if it does, then definitely we'll get in contact. Sure.
Participant: Sure, thank you.
Interviewer: No problem, anything else? So yeah, that's-
Participant: That's all, that's all yeah. I was just curious.
Interviewer: Ok, thank you again.
Participant: No worries, thank you.

Interview transcript 4

Interviewer: Ok, so I just wanted to let you know again that your personal information, such as your name or any uni names that you mention, will all be anonymized. And when we finish the interview, we will send you a recording of it as soon as it's done and we'll also send a transcript, which might take a couple of days. And after you receive your transcript, you'll have two weeks to withdraw your interview or bits of your interview if you'd like, and to do that, you just have to send us an email telling us what you want to withdraw. And feel free to stop me at any point during the interview if you don't want to continue anymore, or if you want to take a break or you have any questions or anything like that.
Participant: Yes, that's fine.
Interviewer: Do you have any questions before we begin?
Participant: No, no, I don't.
Interviewer: OK. So, let's start with, what were the changes to your exams that happened this year?
Participant: Sure, so I was initially slated to have three exams this year and one piece of coursework. The piece of coursework was approximately 1000,1500 I think to 2000 words which was- that became non mandatory and what's the word, summative? Sorry formative, even- formative. Then I had a clinical skills exam, an OSCE, and that was cancelled due to obviously the personal contact that students would have with patients. So that was scrapped. And then we instead had two exams, one based on the knowledge that we undertook or the knowledge we were meant to have studied online. And then a therapeutics exam based upon formulae, medications and prescribing during our third year. So in the end we had two summative exams.
Interviewer: Ok so just want to get it clear in the beginning you were meant to have three exams and one coursework.
Participant: Yep

Interviewer: And that coursework has now turned into a formative.
Participant: Yep.
Interviewer: But you still have two exams?
Participant: Yes, so the clinical skills exam as I said was cancelled, but two exams did remain.
Interviewer: And what was the therapeutics exam- was that one of the two exams that you mentioned?
Participant: Yes, so it was a therapeutics exam and a knowledge-based exam.
Interviewer: Oh ok. So let's start with your two exams. Can you tell me exactly how these two exams were carried out?
Participant: Yeah, so the therapeutics exams, or exam even, has been carried out online - both exams were online. I think this is going to be the most important thing and it follows a rather set pattern. A set training or regimen, if it were, because we've done this exam since at least our first year, but even certainly in our second where this one teacher, this one professor even, instructs us how to prescribe primarily in the dosages, in which case. And the knowledge paper was also online but with additional time. With that given it came to about 3 hours and 45 minutes with 120 questions, one question person per slide and uni-directional.
Interviewer: OK. So both the exams have been turned online and you said for one of the exams you got extra time. Was that for everyone or was that for you personally?
Participant: So actually everyone got extra time, but I got an additional amount of extra time due to my support plan.
Interviewer: Ok and for both of these exams were you supervised, unsupervised?
Participant: Unsupervised for both.
Interviewer: And were you allowed to look at notes, or not?
Participant: Yes, so for both exams we were able to look at notes.
Interviewer: Ok. And can you just tell me how you feel about this change?

Participant: The... overall or just this open book aspect?
Interviewer: So the overall - your exams being online, being unsupervised, and open book
Participant: So, I think, so I think if I'm honest I think that's how it should be. I think most exams would benefit from the aspect of being open book and unsupervised, as I think more accurately, perhaps, it, it represents what we will be experiencing as F1/F2 doctors in- in a clinical setting, and I think the aspects of reliance and interdisciplinary decision-making as a MDT should rely on the aspect that we- should rely on the aspect that we seek input from other healthcare professionals, such as pharmacists, physiotherapists, nurses, etc. So I think the idea of it being open book and online is, is the way forward and to, kudos to my university that most of our exams, if not all, are online for the ease, I think, of marking and data protection and just the collation of data in terms of um ranking, but also in terms of, you could say, for the environment as well as other things so the idea of things being online isn't a new aspect, the aspect of it being open book is very new to us students.
Interviewer: So you've had on line exams before?
Participant: Yes
Interviewer: Oh, ok. Ok, so that way it wasn't a new aspect for you.
Participant: Yeah, it wasn't new.
Interviewer: Ok. And in terms of your coursework now being non mandatory and formative, how do you feel about that?
Participant: Ok, so firstly, the piece of coursework I couldn't complete on the basis that it was a waste of time. It was a waste of time to do it in the beginning, and it was a waste of time to do it during in the middle of a pandemic.
Interviewer: Sorry, before you continue, I forgot to ask, what was the coursework before meant to be like?
Participant: So it was meant to be a, so it's called a community follow up project or CFU and it's designed for students to basically write a report- given a principle such as ethics, economy, health care provided on a given patient that we follow to an extent and we interview and we seek their input from and so it's broken down in two sections roughly where the first section is an almost brief autobiography of this patient, and then the second section is an evaluation of their care.
Interviewer: Right. And before the changes happened, was this coursework into your summative?
Participant: Yes, so before the pandemic, it was not only a summative, but it contributed to our Bachelor of Medical Science and was mandatory to pass this, this, this assignment.
Interviewer: OK, so sorry, can you again tell me how you feel about these changes?
Participant: No problem. So the change of it to change it from formative to summative, I felt was the obvious choice. It…the clinicians and the authorities that be on our course changed it from, from summative to formative on the basis that the clinicians that would mark it or are marking it, are obviously tired out with the pandemic on the front lines and doing a wonderful job there. But I think in reality the piece of coursework itself, being what it is particularly being due after our dissertation that is submitted in January, it's really just a pedantic piece of work in the sense that it really isn't necessary, it really doesn't add any value after you've written the 10,000 words of our dissertation. You know, waited through statistics and what not and cracked that through in about four months to then write this piece that actually doesn't have any contributory value to our degree whatsoever. After I'd then, I, you know, I'd argue when are we going to be writing 2000 words on one patient and evaluating their care? Um, so, so in, so it really…in conclusion, in summary, the decision to transition it from summative to formative, I think, was an obvious decision, but I'd say the most obvious conclusion would be to, be, do away with it, yeah, entirely and replace with something that was a bit more contributory and preparatory to, to our clinical, to our clinical placement that begins in the same month it was due.
Interviewer: Ok, thank you so much for telling me that. Um, that's really helpful. So now moving onto how, um you told me that this coursework use to count towards your degree and now it doesn't. Were there any other changes to the weightings of your exams?

Participant: Yeah, so there was changes to the weightings for exams and what the Med school decided, by democratic votes of 3rd years that are now 4th years, was that the rankings of our 3rd year exams, so the two exams I forementioned will not have any impact on our, our placing whatsoever, and therefore so it wouldn't have an impact- excuse me, let me clarify- it wouldn't have an impact on our BMedScis or our intercalated degree, and as a result it won't have any ranking on our ….on our, on our it won't have any weighting on our ranking overall in medical school, almost like the year has been omitted. And instead our 2nd year grades will be, will be used to, to put us in, give us percentiles and deciles. Particularly for, for obviously our application to foundation programs.
Interviewer: Ok, so um I'm just going to summarize what you told me is that this year has now essentially turned into a pass or fail.
Participant: It, yeah
Interviewer: And the previous year is now counting towards more than it used to.
Participant: Yeah, so and sadly things are a bit up in the air at the moment even now, but what the current standing is that the average grade of our 2nd year will be the one that's added to our, to our dissertation, and that will make our degree ranking, our degree grade or class. Additionally to that, degree aside, it will also contribute to where we uh rank in medical school for our Foundation Program. So yes, it has just become a pass or fail with no impact on the year coming, so our 4th year, or on the year below, i.e. our disserta- i.e. our degree.
Interviewer: Ok. Ok, and again, how do you feel about these changes that happened?
Participant: Well, firstly again, I I think it's important, I think it's- firstly, the idea of it being democratic, so up to the students to decide I think was very smart. I think that avoided a lot of backlash towards the medical school, in case they did go authoritative and make a decision on their own, so I think that was-that's a good thing. I personally voted that our 2nd year grades would, would contribute and omit instead our 3rd year. But I also think that third year, I don't think really there should have been tests at all, and I think that our or my University have a habit of hiding behind GMC guidelines and, and protocol that you know the average medical student doesn't obviously know. We don't really have any impact into how the GMC evaluates the University. We don't know the system, and I think we, we find ourselves simply saying yes, ok, we agree, we don't have a choice, let's go along with it. And you know, we look at other universities that didn't examine their students or gave them a formative exam or simply told him to be well and, you know, wish them the best during this, this horrible pandemic. And not just the pandemic, but obviously the many things that the pandemic has been a ripple effect towards and the simultaneous things that obviously happened concurrently in the US and many many other countries like Australia. So, so I think in reality- it's good that they had a democratic choice, but in reality, but truthfully the exam shouldn't have existed in the first place. I don't see any benefit from examining students in the middle of pandemic when they can't, they can't allow an equal playing field for the numerous people- accounting for their countries, given personal circumstances and the impact that it's had on their, on their, on their personal lives, particularly when, in the UK at least, tests- COVID tests- are simply near to none for students, let alone clinical staff, so I see no benefit whatsoever to testing us.
Interviewer: Ok, thank you for that. [broken up] It's really helpful for us to hear these opinions. [inaudible speech]
Participant: I'm sorry I didn't quite hear you there.
Interviewer: Sorry, I was just saying thank you for telling me that again, it's really helpful to- for us.
Participant: Sure.
Interviewer: So I'm going to move on to… your ideal exam type so before this whole pandemic happened, what would your ideal exam type have been like?
Participant: I think before this pandemic, I think in terms of the OSCEs firstly, if we could break it down into like, into clinical and nonclinical. I think the OSCEs are absolutely fine. I think they're really important. I think they give the right amount of pressure for students in which they've obviously time limited and as we progress into our clinical years, they give us the, the, the added I think bonus of not just seeing normal, quote unquote normal patients but also those that have some type of underlying

pathology or, or overt pathology and allows us to investigate that. Of course, on a variety of genders, sexes and skin tones and so, although I think the variety of patient is lacking, I think that's maybe a slightly different issue, but overall I think clinical skills are well, but I think they could, we could do with more people of colour as, as patients to inform our understanding. If we were then to move on to non-clinical, I do think that open book is the way forward. I think it's about using the resources available to us in a logical manner, not just to help us as tomorrow's doctors, but also as, as today's medical students - and why do I say that, I say that because it impacts the quality of care that we can provide to our patients if we are familiar with trust guidelines, if we're familiar with the BNF app as opposed to the book, if we are able to communicate better and understand the roles of, of sisters, nurses, hospital managers rather than simply relying upon our own quote unquote Intelligence in order to solve a problem that fundamentally wouldn't be solved with us alone. So it sounds really counterproductive to me to train today's students to be tomorrow's doctors and actually under prepare them or underutilize their skills that, you know they've so thoroughly tested and expected from us in our interviews in order to get into medical school and actually not in order to, not actually give them the resources properly available. I think that's rather- rather ironic, so, so I do think that in my opinion would be open book. I think there'd be more, more more tests, and I say this because the University of X actually had modular exams and then they changed it when I went into my first year, so they kind of brought all that testing, all that examination right until the end and why I probably, what I'd do in my opinion, is that I'd have more case based learning that would more severely, not severely, more, better critique the aspect of maybe an MDT or real patient. And I think in terms of – and I say that because that is fundamentally what we are going to be facing and it, and to give an example of this, there's been about four or five times now, I've gone onto the wards, now that we've obviously restarted. And I'll be talking to an F1 or F2 and they'll simply say that's not done here. Then you know, I'm there thinking, wait, that's what I've been examined on, that's what I've been taught, that's what I've been told happens, and they're like, no, we don't actually use that drug for this, or we don't use MONA for acute coronary disorders, actually. Instead, we're moving away towards this. We're moving towards that. And you know, I think that actually what we could do instead is focus more on the clinical skills, the bedside manner, the cannulation, the venepuncture. And focus, and, and with that you know, bring in prescribing, which is such a huge aspect of Medicine, because obviously, you know, I think we're one of the very few professions that can prescribe, although that's- prescribe medications, although again that's changing. But instead bringing more, more- it's basically effectively, bring in the more practical aspects of what we do and shun maybe more of the theoretical, non-practical, dare I say, less useful aspects instead, and that I think, starts by having, by having, dare I say, yeah, by having an open book exam. And that, that can allow harder questions which were present in our in our knowledge paper, that I've again just- already mentioned, because it made us think more laterally- it said, if Bob has these set of symptoms, he's been on this type of drug, you know, as a first line medication and then not working, what would you suggest as the second line of drug, you know? And that's the type of questions that I'd like to see. That's the type of questions that my seniors, my consultants, my F1s are asking me in the hospital, and those are the things that are really making me making me think, as opposed to the, you know, the physiology. Also, if you need the biochemical pathway of aspirin or whatever, it may be- well, actually by the time of F1, I don't really remember that. You know, even now in the 4th year, I'm not really interested in the biochemical pathway of aspirin. I'm just learning it because that was expected of me.
Interviewer: Yeah, thank you. Yeah, that was very insightful. So you're saying that your ideal exam would be online and more exams throughout the year focusing more on the clinical aspects of things, if I summarize what you've said so far.
Participant: Yeah, I think Medicine can be pushed more towards a, a rigorous apprenticeship type of course than what it is currently.
Interviewer: Ok, um and if you were to choose how exams should have been carried out right now, in this- during this pandemic, what do you think should have happened?

Participant: So, is this on the basis of if I had to have had exams, how would I have wanted them to be like?
Interviewer: It's not that you need to have exam, it's just what you would have preferred.
Participant: Ok, so yeah I would have preferred there wasn't an exam if I'm honest or if there was an exam it was purely formative to gauge where we were at. If it were, where-what our standard of learning was. And that would therefore inform the University, the medical school how to, how to teach us, what our shortfalls were, what our strengths were in order to better prepare us for, for the coming clinical years.
Interviewer: Um and in terms of weighting of things, since you're saying all exams this year should have been cancelled. Do you think weighting of exams from other years should have stayed the same, increased, decreased?
Participant: Well, I think, I think they, they could have stayed the same or they, they could have increased. I think so- it depends what year you're in, at least at the University of X you see because like most universities our first year doesn't count, but our second year counts towards our BMedSci, our third year, we have a degree, a dissertation, even, that is the technical finish of our BMedSci and we have to finish a clinical placement of six months which we call CP1 which also contributes to our BMedSci. So when we get to 4th year it has no impact on our dissertation whatsoever. We've got our degree. We've been, we've graduated. We had our graduation party, etc. And that's how it goes. So I do think it depends on what year you're in in terms of how it's weighted. I think that for the University of X in what they've done so far, I think it's the again the logical choice in saying, ok, well, we can't- we can't - you can't really quantify on two exams how someone did, particularly when you omit the vital aspect of clinical skills during a clinical placement, and we know that people presumably tried their best in the second year, when obviously COVID wasn't necessarily a thing, so we're going to take, take them. Now for the year above us, for the, for those that are now 5th years, they didn't have any exams at all and instead what they did is that they they've moved, the, the 4th- excuse me- the 4th year exams into a month where they're going to have their finals.
Interviewer: Oh ok.
Participant: So that's going to be a really really stressful month of, these, for these students and some of my friends are in that year. Now I think if we're going to talk, if we're going to talk theoretically as if I was in the year above, I think they should have spread those exams out a bit more, or at least allow them to be tested in part, if that makes sense, so you know we're going to test 10% of this exam now on this aspect and stagger it in preparation for their final exams, because they've obviously missed out a huge chunk of, of their, of information. Yes, they have some clinical experience, but they haven't had the, a lot of the surgical experience as, as it's at least structured at the University of X and, and a lot of the clinical experience and specials, or specialties rather, as we, as we call them in in X, has also- has also been omitted so basically what I'm saying is, I think if I was, again if I was to talk as if I was a fourth year, I think the University of X should utilize the exams in order to prepare their students for their finals, acknowledging that they've missed obviously huge degree of their, of- a huge degree of their information in the build-up to what normal students, normal medics would have and obviously be fully prepared for, so that's what I would do, I would almost make the exams modular. They'd have finals in May as I think they are scheduled to be. And they would have these exams in, let's say, January, for example, and if they obviously did not pass them, they would then have the opportunity to retake them in the summer. But fundamentally, these exams would allow them to say by January, I should have the knowledge of 4th year that I obviously didn't get, because I, you know, of, because of the pandemic and that means I know that if I pass this exam at 40%, 50% whatever they set the grade boundary at, then I'll be ready for my fifth year in May. And that's personally how I'd do it and obviously because they'll be taking this exam, they know that they are at the level at which the GMC predicate them to be.
Interviewer: Ok, yeah. Thank you for that. I just wanted to ask you about your preference again, because I wasn't quite clear. So between in hall examination and online examinations, which one would you say you prefer?

Participant: Well, I think, to be honest, it- I think it depends. Um, and I know that may not be the correct answer, but I had a resit on Wednesday for example, and I had a paper examination. Now the paper examination's really- it was useful, but I can see how mistakes could be made or an examiner could say, you circled C but then you rubbed it out, but you also circled A, I wasn't sure which one you chose. So, I can see how mistakes could quite easily be made in a paper examination, as opposed to a, a online examination. And also, I think there's a degree of security, there's a e- as I think I've mentioned, there's a degree of data protection, there's a ease of marking, obviously, in terms of the labour, again particularly during a pandemic so I think overall I I'd lean towards a- a an online examination. I think the aspect of halls is almost redundant in a way because if someone is taking an online examination, it doesn't mean that, obviously, they're- not- in an environment that is secure. If we were to, to side with an exam that, that had to be non-open book or entirely you know, knowledge based if it were, so I, I don't, I don't, I think, yeah, I'm not sure.
Interviewer: Ok
Participant: If in halls is ever going to be thing.
Interviewer: Yeah, um ok, let's move on to the next question now. Um, I just wanted to ask you, for you personally, what are the most important factors that make an exam satisfactory? And when we say this, we're not really talking about the contents that it's assessed on, just the exam itself. So, the environment or the time, or you know, things like that, [inaudible speech].
Participant: Sure sorry, could you just say that first sentence again? You said what are the most important factors an exam should be assessed on and then it kind of dipped a little.
Interviewer: Sorry, sorry I will repeat the question again. What are the most important factors to you for an exam to be satisfactory, and we're not talking about the contents that the exam assesses, just the exam itself.
Participant: Hmm, just the exam itself. So, so I think, ugh I guess there's a lot, lot in there, but I, I'd say the first thing is that obviously you want it to be uniform as possible, which is standard as possible, and I've obviously spoken of open book exams, but I truly believe that if someone wanted to, for example, go into a hall as per your previous question, that actually be an option as well. And I say that because a lot of my friends and even myself had technical difficulties while at home, and I know a lot of my friends elsewhere within the UK or abroad had poorer Internet connections than they did when they were at university for example. As is necessary, at least for Rogo examination. platforms in order to do the exam. So I think that there should be an option that someone should be able to go in halls, in halls because and this is a very real thing that sadly one of my friends actually had her Internet go down during her exam and then technical difficulties on the University side, on the software side that prevented her from continuing once her Internet got back up after I think a couple of minutes, and what that basically means that she failed the exam and she can't progress to 4th year without it, without that pass. But to think about, let's say a month or so after the exam we had a two-week break. Sorry excuse me, we had a two week break after the exam and then we started 4th year so we had effectively a two week summer in the midst of a pandemic and everything that was going on, started 4th year and we've still had to study for this exam that was from our previous year while performing examinations in our, in our current placement called mandatory assessments with clinical skills and obviously preparing for our exams next year.
Interviewer: Sounds stressful.
Participant: Yeah, it's aha incredibly- incredibly stressful, and to add to this we didn't know when our exam was. We didn't know the time, we didn't know the format and we were just informed a week before as to the time it will be and the format it will be in. And so we didn't know if it was going to be open book like, like our previous exam, [clears throat] - excuse me - in the summer, so in June ish. And we didn't know if it was going to be online as was again the previous exam. Instead it was actually- it was closed book. It was on a physical piece of paper as I- I think I mentioned because of the mistakes that can occur and- and actually obviously there was a degree of COVID precaution as well, which is also quite daunting. You know when you're going into an exam, you shouldn't have to sign a declaration that says, you know, are you a risk to other people, and you know if you are a risk

to other people, what do you do? (laughs) Do you just skip this exam when you know that you can't pass- you can't progress to 4th year without it? Having, you know, ironically, having already started 4th year- we've been doing it for three months, and then there's obviously implications. Implications financially. Of course there is, in terms of resources, because the NHS bursary is already been paying you the, the Student Finance England has already been paying you and you've really been getting tied into to your bursaries and whatnot, so you can't just back out, you can't even take a break in , in terms of if you were, if you did have extenuating circumstances, safety issues, and heaven forbid anything else, so, I guess. I guess that, you know, that long story short in this regard, firstly, I think I'm really passionate about that there has to be a standardized way in which people are examined in the physical environment. If someone in, you know, let's say we have campuses in Malaysia, I think so. If someone in Malaysia, for example had - had great Internet but someone in China where we also have a campus, had extremely poor Internet, then that needs to be factored in. If, if we – yeah?
Interviewer: Sorry, sorry for cutting you off there. How do you suggest these changes be made?
Participant: So I think the first thing we need to we need to say is that actually if someone wants to still have a physical examination, i.e. come into a hall as you mentioned previously, then that HAS to be an option. I think, so yeah?
Interviewer: So I'm just trying to understand exactly what you said- so you meant, um, people should have the choice to do it online or in a hall, the same exam?
Participant: Yes.
Interviewer: Ok.
Participant: I think the second thing would be, you can obviously do an Internet speed test and whatnot, so I think that someone shouldn't take the exam or should be at least pre warned regarding taking the exam- um let's say that they should be *cautioned* about taking an exam if their, their Internet speed is below a certain amount.
Interviewer: Yeah
Participant: OK, because we had, you know, I had, you know I think, oooh, you had about just over a minute or so per question, ok. And when you're obviously reading a case study, which it was it was and reading the sentence, you're like, Oh my gosh, let me think, you know, what this- what does this correlate to? What do these bloods correlate to? What do these U and Es correlate to, LFTS, TFTS etc? And then obviously you've gone open book, which obviously, potentially researching this information- actually it's kind of useless. It's actually more of a distraction to have this open book as compared to, as compared to, to simply just answering the question closed book as per our previous exam, so I think, yeah, so – (laughs)- so basically the Internet is going to be a huge problem. We would obvious- I think there's an aspect of, obviously, technology, but that's somewhat out of a student's hands. That's again on the base of the University, so I'd expect them to obviously run diagnostics, to ensure their software is reliable, to ensure that someone's on hand to, to answer any questions should something have gone wrong and other precautions I can only presume are out there such as auto saving of an exam and all that good stuff. So that's the second thing you know, there's the first thing that there should be a standardized environment as far as obviously physically possible. There should be a, a support on hand and then I'd see the third thing is that the timings should be fair. You know. I think a lot of people have special circumstances. A lot of people like myself for example, I can only talk for myself, but I can talk, but I know other people, you know, they didn't have an environment which they could take an exam right? When they lived at home they were surrounded by their siblings, their parents, even their partners and, and I know that for me, taking an exam for 4 hours for my partners was quite disrupt- disrupted for her because she, she worked- she was working from home on, on the Internet on her, on her computer and whatnot as well. So again, you know , that could be solved by having, by everyone having the opportunity to, to study- sorry, not study, to take the exam in a hall in a standardized environment provided by the University. But. but I think that, you know, there- there just has to be some type of compromise - or not even compromise-some type of understanding that, that these- during a pandemic that people don't have the same resources. People don't have the same access. I, I went to the University campus

for example. I went to all three or sorry, three of the university campuses and I got kicked out from all of them. And then I was told that if I was caught there again, they'd take my student ID card, but I didn't have a laptop. I didn't have Internet access. My course was entirely online and there was no, dare I say, mercy or understanding whatsoever, and so not only was almost fighting at the University to an extent to get a loaned laptop and basically get these resources that we'd normally have, should it be a non-pandemic and I know that University employees were having loaned laptops, but also I was on the other hand fighting the aspect that I literally can't access my course without it, I literally can't access my course without a laptop. So literally what you want me to do? This is despite being threatened to almost have my studentship revoked, so I guess that's the third thing is that there just needs to be some degree of understanding that whether it be, again, a non-pandemic, or if this was taken into the future that there- people don't have the same resources. People don't have the same opportunities around the globe, and of course, even in the UK I think there's again so many to mention, and I think we're just running a bit overtime, but there- there has to be some type of, of compromise or understanding there.
Interviewer: Yeah. Thank you for that. We are running a bit short on time, so I'll move on to the final question now. So final question is: how was your confidence going into this exam compared to how it would have been previously?
Participant: So going into the, so if we speak of the exam I took in in June, I actually had to make a decision, right, I had two exams, etc. Therapeutics based one and knowledge base one and I said to myself, I need to at least pass one of these exams, you know I can't be resetting two exams, and it got to a point where I haven't had my laptop for about two months, three months, and I've been -it's- really ill and stuff like that and other things had unfortunately occurred, and I said, again, I need to pass at least one of these exams, at least that will alleviate some of the pressure should I need to resit one of them and so I thankfully passed the therapeutics exams, but as I, obviously, as is obvious, I failed the knowledge exam and, so, in terms of confidence is- there was none if I'm honest.
There was just very much, ok, I know what the therapeutics one wants from me, it's a set standard, I've done this before. Obviously, it's going to be elevated in terms of its difficulty in account for it being my third year, but I I I somewhat know what they expect from me. And in comparison to the knowledge paper, actually the knowledge paper was nothing like what our mock papers were like. Nothing like what the previous exam was, in fact it was much, much hard. In fact, you know we had some of the lowest grades ever, not just for the- not just for our year- in- as a cohort, but in the University of X categorically because it was so difficult, and I think you can obviously factor in the circumstance for it, the circumstance in which it was taken as well, so- so my confidence was, was very low in comparison. And to compare this further, the resit I just took on Wednesday although I had been balancing numerous other commitments, personal and professional. Of course 4th year being one of those, and the exams that I've been taking weekly. Actually, my confidence was also a lot higher and I feel a lot more prepared at having given- having been given the extra time.
Interviewer: OK. Um, yeah, I mean, you mentioned that you had a lot of different circumstances leading up to the exam that did affect your confidence. I mean, feel free to talk about it. Again, you don't have to if you don't want to. It's up to you if you want to go in more details about it.
Participant: Sure, sure, so I found out that my partner is pregnant, which has been amazing.
Interviewer: [inaudible speech]
Participant: I had - thank you, I think you said congrats, but I couldn't quite hear, but thank you.
Interviewer: [inaudible speech]
Interviewer: I had physiotherapy, I began therapy, I - as I said didn't have a laptop for a long time, I had- I was volunteering, in fact, in my community because a lot of people I was surrounded by in X didn't have a lot of food, so I was a volunteer delivery driver during a lot of that period and, and actually just think my mental health was very, was quite, my mental health just deteriorated really, in lockdown, particularly because I think, I think, I have- I don't think, I have learning disabilities. Is that something that is politically correct? I don't even know and I have them, and, and just being indoors and not having the resources to study was, was suppressive. It was really difficult to handle,

particularly, with, as I think I've implied the aspect, the, the fractitious nature of of the University of X in terms-in regards to, at gaining access to things that are, that are necessary. So again, loan laptops that are normally provided and providing proof of various things, that obviously weren't actually available, simply because the health care providers were closed, and so that – it was just a tremendously difficult time with these [inaudible] as well as some familial issues that made things extremely, extremely difficult and, and in fact- actually I forgot, like we got evicted as well, we had to move, in- in August and we were told we had to move a bit earlier, but we fundamentally moved in in August simply because the previous apartment we were in, actually was, was falling apart and was leaking and stuff, so there was a lot going on in a very short space of time. And- and again, that affected my- my work.

Interviewer: You told me [inaudible speech]

Participant: I'm sorry I can't quite hear you.

Interviewer: Sorry um, can you hear me now?

Participant: Yes I can.

Interviewer: I was just saying from what you've told- told me so far, it seems like you've had a very stressful period before the exam. Yeah, really sorry to hear that. But thank you so much for telling me that information, honestly it's very helpful towards us towards our study. Thank you. I just want to end this interview with, I have few more questions. Sorry, it is going a bit overtime.

Participant: Oh it's fine.

Interviewer: Do you have some time to talk about this couple- 5 mins max?

Participant: Yes that's fine

Interviewer: So again you did tell me about the circumstances leading up to the exam that decreased your confidence level. But when it comes to the exam itself, so things like it being online and open book, did that have any effect on your confidence level?

Participant: I think it did. Yes, as I said, the exam was not, the exam wasn't related to the previous questions that we had, and so excluding all the other circumstances, some of which were obviously amazing like, becoming a father. So it's not all bad, but the aspect of, of it not being consistent with previous exams, with the- with the aspect of not having mock papers to utilize, for example, because again, they changed our- our cohorts examination style. [coughs] Sorry, excuse me- and therefore the previous cohort, i.e. those that are now, now in their 5th year, are unable to, to give us their, their notes and, and, and mock papers, so it doesn't- it doesn't correlate, so I think that is not just again my personal circumstances, I mean it's on the aspect of the University. They weren't testing us in a uniform way that was, had- that had been tested previously, both structurally in terms of our cohort being different, and also in the aspect of- in light of the pandemic they made the exam harder in order to compensate for the open book aspect. For example, I, I took the exam. Again, as I resat it on Wednesday and having resat it on Wednesday - one, as I said, it was far more closely related to the, to the weekly exams they gave us. Two. It was only 80 questions as opposed to 120 and Three. It wasn't as- it wasn't as convoluted, it wasn't as - it focused more on our learning objectives effectively rather than trying to compensate for the aspect of it being open book. So I gave the example earlier that you know Bob had this drug, so Bob had these symptoms, he was given this drug. What's the second line? And you know, Bob may have a contraindication, or allergy or something like that, and they will have to think of you know, not just the second line aspect of the treatment, but also the, the alternative second line aspect, given the allergy or given the fact they didn't agree with a certain medication, so that was very much what it was like, and again, if we focus on that periodically, categorically, uniformly throughout the year, I 100% understand. But unfortunately I'd say we didn't- the University didn't teach us to do that. Instead, what happened was is that in- at least in the University of X, where our learning objectives are always structured to the, to the extent where in second year where- So let me start, first year we're learning diseases. Second year we're learning the complications of these, third year we're learning about treatment forth year management, and then fifth year, basically, the combination of all of these skills, obviously in preparation to become a doctor, in in preparation to our finals. All of those aspects accumulate to that point, and what they

were testing us in, in in 3rd year was officially what we were learning in fourth year. With these management aspects, these complications, these, such as, such as allergies and stuff like this, was really actually way beyond our breadth of knowledge and you know, by and large I haven't spoken to a single medical student that has said, actually I was very well prepared for that exam. Actually, I felt very well established. Actually, we were taught this. There were things that came up in the exam that we had never seen before, right? We were using PowerPoint slides and lectures from 2007, 2009. You know, over 13 years ago, with the assumption that the NICE guidelines hadn't changed, that to me is absurd, not just as you're training medical students, because obviously, you know, we got a BNF that changes every six months, for example. But because obviously the NICE guidelines had in fact changed. And what was the point in us being examined on these antiquated procedures and protocols when literally two months down the line, we're going to be in a hospital in which we're going to be bombarded with current trust guidelines and, and NICE protocol. So, you know, it just, I just think it was it was rather ridiculous. It was rather ridiculous. And personal circumstances aside- they obviously played a huge aspect, but in fact we, we could just be - we could have just been better taught. You know, we could have had a University that actually had an infrastructure that had things online in the first place, that recorded their lectures periodically to ensure that they were up to date or even consulted the NICE guidelines would have been - would have been a bonus.
Interviewer: That's all the questions I have for you today. Thank you so much for your time. It's been really, really helpful for us. Before we end, is there anything you'd like to add to what you've already told us? Any questions you have?
Participant: No, I don't think so. I don't think so. I think I'm no- I appreciate your time. I appreciate you doing this. I think this is so so important, again with things that are going on it's likely that- it's likely that this this environment, shall we say this, the ramifications of COVID will extend to next year at least, if not the year after. So I do think that this study is so important and appreciate yourself and your colleagues are doing this because I think it will benefit not just the average student but also the University as well.
Interviewer: Thank you and again thank you for your time. Yeah, so I just wanted to remind you that as soon as we end this-I end this interview. You should get the recording and the transcript in a couple of days which feel free to have a look at it. And if you want anything changed just email us.
Participant: OK, thank you very much.
Interviewer: Thank you so much for your time. Yeah bye.
Participant: Bye. Take care.
Interviewer: You too

Interview transcript 5

Interviewer: Ok, so the recording's just started, so hi, I'm Anusha, one of the student researchers. So, today, I'll just be asking you a few questions. The interview is being recorded, and you'll get a copy of this recording immediately after which you can look through if you want to and will send you a transcript of the interview, and then two weeks after you get the transcript, up until then you can withdraw any information that you want, or you can withdraw the whole interview if you'd like to. So, have you got any questions before we get started?
Participant: Uhh no
Interviewer: OK and just to remind you that you will be anonymized, and any mentions you make to University names or people will also be anonymized.
Participant: Yeah, sure
Participant: Ok great. So I think, if you don't have any questions, we can get started?
Participant: Yeah. This is making me nervous, god. [laughs]
Interviewer: [laughs] Don't be nervous at all, it's just a casual chat. I have to say all this stuff just to make sure we do everything properly, and yeah, if you want to stop the interview at any point and if you would like to skip any questions or anything just let me know- like feel free to like- if you want to take a break at any time.
Participant: Ok.
Interviewer: So, can you just like tell me a bit about what the changes were to your exams this year?
Participant: Changes to our exam, so first of all, so I'm currently in fourth year, but the change affected me when I was in 3rd year. Exams are normally- we normally have a clinical exam, which is an OSCE and also a written paper. However, due to COVID, the clinical exam was cancelled and the written paper was converted into an online multiple choice exam.
Interviewer: OK, great, so you had an online multiple choice exam.
Participant: Yep
Interviewer: Could you tell me a bit about how exactly the online exam was carried out?
Participant: So um how the online exam was carried out. So it was done on this platform called - can you remember what it's called? Hello. Hello. Hello?
Interviewer: Hello, hi yeah, sorry I couldn't hear you for a bit, can you hear me?
Participant: Yeah, sorry my connection's a bit unstable so it was done- yeah, so it was on- so it was an open book exam, and students were given this platform, which I forgot the name of, can you remember the name Anusha?
Interviewer: Um, it's fine, just carry on.
Participant: Yeah students were given a sort of passcode before the exam and then when the time comes around like half an hour beforehand, you use the code -around 15 minutes before -you use the code you log in, which would then load up the platform which contains, I think, around 150 SBA's to do online. The exam was open book, so we had access to Google and everything. And the questions were mostly clinical scenarios which then asked so as a- the questions normally asked you for sort of clinical- which assessed clinical reasoning etc.
Interviewer: Yeah. Ok thanks that's really helpful and what were your feelings about this exam?
Participant: So I guess initially, I was slightly unsure, because, first of all, not many exams have been done online previously and also with the fact that it's open book, I wasn't sure whether or not the outcomes of the exam would be discriminative and representative of - of the students, sort of how much work they put in, their capabilities. However it ended up being like-it turned out- turned out really well so the exam went really smoothly and the questions - so the way that the exam that the faculty set exams was that the questions were difficult for students to Google. Despite the fact that they had access to Internet, it was still difficult to utilise the resources. So, it ended up being- I felt that it was a good- it went well basically. It was above expectations. [laughs]
Interviewer: Ok that sounds good. Could you tell me a bit about the changes that happened, if there were any, to the weighting of the exam? So how it contributed towards your degree?

Participant: Um, oh God, I don't think a lot of information has been announced yet about the weighting of my third exam, so before I believe that the OSCE and also the written paper both contributed to our FPAS applications. But because the OSCE has been cancelled now, I don't - I don't think that they've announced anything yet about the weighting. I might be wrong. Sorry, wasn't very helpful there
Interviewer: No, no, it was helpful, don't worry! And was there any - do you have any other information about the weighting, so for example, whether it was pass/fail or summative or formative or anything else?
Participant: Uh so. I know the exam is summative so it would definitely count towards the-the actual FPAS scoring. It was also pass/fail, yes, but students are also- the, the top 30% of the students are also given sort of distinction star for the top 10%, distinction for the second decile and merit for the third decile as well so but it was- overall it was a pass fail exam, yeah.
Interviewer: OK and how did you feel about this?
Participant: Um, it was fine for me because that's what it's always been like, and I personally felt that the on- the fact that the exam has been switched to an online format did not- it was, it was still representative of a normal exam. It was similar to a normal, um, exam I guess. Obviously I know that some students may be disadvantaged if due to some reasons that like, at home and things like that, but for me, I thought overall it went well so yeah, yeah [laughs]
Interviewer: Yeah, I see what you mean. And you mentioned not having much information about like how it contributes towards FPAS. What are your thoughts about this?
Participant: To be 100% honest, I actually haven't thought much about this until today, but this actually reminds me, so it, it- I guess it would be nice if the faculty could be a bit clearer on the sort of the sort of future and where this is going, but obviously I understand that there's still a global pandemic going on, and they're very busy right now with the new students as well. With the University going back to, sort, of going back to normal, like restarting. So I understand that the faculty will be busy. And I guess it is annoying as a student that you are kind of in the blind a little bit, but I guess I'll just be patient and wait, so yeah.
Interviewer: I see what you mean.
Participant: Sorry, it's really, my- my answers are really ambiguous. [laughs]
Interviewer: No, no, not at all, it's like what you think
Participant: Yeah
Interviewer: - and that's all we want to know.
Participant: Yeah
Interviewer: Like it doesn't need to be a strong opinion either side.
Participant: Ok, ok, sure.
Interviewer: Ok, is there anything else you'd like to add about the changes to your exams and how you felt about them?
Participant: So, uh, I think -so obviously I know that the OSCEs were cancelled because of safety reasons etc. But I also think that's kind of a- it's a bit of a shame because I feel that the OSCEs- the fact that it's cancelled, so a lot of students then therefore would perhaps prioritize other things such as the written paper over the clinical exam, which I think will be a big problem. Because well, personally, I feel that I'm not very clinically competent because- because of the OSCE being cancelled. So I- I personally put more focus on the other exams and- so it does make me slightly ***worried*** for example, when I, when I- when I reach my second clinical year, which is in fifth year, will it be difficult for me to pick up these things, like again and etc? But, yeah.
Interviewer: That makes sense. That's like really helpful to consider. And anything else you want to add?
Participant: No, I think that was my main sort of concern.
Interviewer: Ok, and just to check you didn't have any other exams apart from your OSCE and that written exam that you had scheduled during that time?
Participant: Oh yeah, during that time, it was just the online exam.

Interviewer: And what was the environment that you carried out the online exam in?
Participant: So I did the online exam at home. My house is relatively quiet so it was fine for me, so I understand that some of my friends, and some, some people I know who have a busy household which could then – which could have maybe made the situation a bit worse?
Interviewer: Yeah, and do you have any other thoughts about the exam environment and how it changed to how it would have been and what you felt about it?
Participant: Uh. Yeah, so… actually. I guess I think the exam itself, it was fine. So the fact that was online and things it, it ran smoothly, but the fact that students had different home environments or perhaps different residential environments where they carried out the exam could then perhaps could have led to slight disparities, which may, which may mean that their performance was affected by the environment. So yeah, it's- it's a factor, which is very difficult to control.
Interviewer: Yeah, yeah. OK um, feel free to add anything else on that topic. But if it's ok with you, I'll move to the next question?
Participant: Yeah, sure, sure.
Interviewer: So what would your ideal exam type have been in these circumstances that we find ourselves then?
Participant: So during the middle of the pandemic?
Interviewer: Yeah during the middle of the COVID-19 crisis.
Participant: I personally think that, if an exam was going to happen, under these situations, then what we did would have been - was the best they could have done. It's either that or you cancel- oh I don't want to be so like- but obviously the ideal situation would be do it in an exam hall but that's not possible, and, I can't really see any other ways of doing during the exam under a safe sort of environment whilst also maintaining a little bit of… yeah, yeah, you get what I'm saying.
Interviewer: Yeah so just to like double check, so you think the exact ideal exam type during COVID would be an online open book exam.
Participant: Uh, yeah, yeah, because you can't really, you can't really- you can't say closed book because you can't really control that, so might as well just make it open book-open book and yeah.
Interviewer: Ok and you mentioned that in an ideal scenario if there wasn't the pandemic happening your ideal exam would be in an exam hall? Are there any other things that you think that you think would make an ideal exam in a non-pandemic scenario?
Participant: So obviously an environment that's quiet, obviously, so, to allow students to focus and also an environment that's easily sort of accessible by most students so then- so that most students can have- so then there's consistency amongst the year group for the students who are taking the exam. Yeah, yeah, and obviously an environment that's controlled so there's no sort of cheating and things like that.
Interviewer: Ok. That's really helpful and kind of leading on from that- similarly, what are the most important aspects that an exam should have for you, for it to be a satisfactory exam for you, or for students in general?
Participant: Um, let me have a think so…
Interviewer: Yeah, take your time.
Participant: Is this just for the exam paper or like including the environment as well?
Interviewer: It can be anything?
Participant: For, for the exam itself, an ideal exam for me would be an exam that's- that's obviously assesses content that we were taught, but also contains questions that can - contains adequate- an adequate range of questions which can help the teachers, the lecturers, the faculty to differentiate between the students. And also in terms of environment, I think it uh- Oh yeah, also, the **format** of the exam, I think, I, I quite like the current University X, so I go to University X- oh you're going to mute this so wait - so my current exam format is that I have SBAs and also long answer questions. So I think that's a good balance for an exam as well because with medicine, there's a very wide range of content that needs to be tested and it's impossible for the faculty to test everything using long answer questions and therefore SBA's need to be implemented as well. But it's also important to

have a good balance between SBAs and so short answer, long answer questions to assess deeper understanding in the students. So that's for the exam, for the environment, I've pretty much mentioned about the environment earlier, so somewhere that's quiet and things like that, yeah?
Interviewer: And are there any other things that you think are important to consider, irrespective of the contents of the exam, to make an exam satisfactory, apart from the stuff you mentioned already.
Participant: Umm…
Interviewer: If there isn't it's fine, just if you can think of anything.
Participant: Yeah um I guess something that's- I guess it's something that assesses- as in it assesses the skil- So for medical students in an exam, it's important that an exam matches with, for example, criteria of the GMC, things like that to make sure that our required competencies are met, so yeah.
Interviewer: Ok. And is there anything else you'd want the people setting your exam to have in mind when they were deciding what kind of exam they should implement?
Participant: As in, during COVID?
Interviewer: Um you could, if you have different answers for during COVID and in general, feel free to tell me both.
Participant: Sorry, so the question is- anything that the examiners should have in mind when they're writing exams -is that the question?
Interviewer: When they're kind of like deciding the format of the exam?
Participant: The format of the exam. Um, yeah so… I think. You want the exam to be under a controlled environment in order to make it fair and representative and so, um, therefore, in person exams in an exam hall etc would allow that, sort of a, control element. But I understand that of course, like during COVID things uh- it will be difficult and so yeah, I think - I think that that's the main thing really. Sorry, I can't really-I feel I've said everything, like before.
Interviewer: Yeah that's really good, that's super helpful. Do you have any ideas perhaps on how you would ensure a controlled environment during COVID?
Participant: So um I'm aware that like some universities have implemented a technique where – well not a technique- they, they get an invigilator to observe a group of students whilst they're taking the exam through Zoom. So that that's -that's something that they could do. Obviously it's still not ideal because it's- it's not like perfect control. But it's, I guess it's the best you can do really during these difficult times.
Interviewer: And if you were to compare the control situation you just described with an unsupervised situation, which one would you prefer?
Participant: I think- so that really depends on what exam it is so.
Interviewer: Ok.
Participant: Because nowadays with Internet being so- um- actually, if I guess like if, if, if the method I mentioned - having an invigilator on zoom is implemented. I guess that can only control for things like if students look at textbooks or something, but students will simply -there, there are ways to get around that, but yeah, I guess I will prefer that over complete open book.
Interviewer: Ok, that makes sense. Do you have anything else to add?
Participant: No.
Interviewer: So we're just going to go on to ask about how- what was your confidence going into the exam compared to what you think it would have been otherwise, like without the COVID-19 crisis?
Participant: So I do feel like I felt safer and more confident because, because of access to Google. [laughs] Yeah, so definitely it felt better and because having the home environment as well, it was familiar so that was nice as well.
Interviewer: Ok, and was there anything in the circumstances leading up to the exam that affected your confidence?
Participant: Not really. I guess- I guess the common things like you hearing peers , like how they revise, and you get stressed and things like that. But that's like the classic stuff. But yeah, other than that, not really no.

Interviewer: Yeah, do you think that was any different to how it would have been without the COVID-19 crisis?
Participant: Not really, no, not really. It's just yeah, it's just a culture. [laughs]
Interviewer: Yeah, I understand. Anything else that you think could have affected your confidence?
Participant: Um, I guess technical problems like, if you know that you have a really dodgy Internet connection, then you could be quite worried on the day of your exam because for example, something like this, which contributes to our future job applications, you kind of want to do well. And if you have a dodgy Internet or something, or your computer breaks down- basically access to sort of your equipment, etc to run these online exams I guess. That could affect -that *could have* affected my conference.
Interviewer: Yeah, yeah, that makes sense. So I'm just going to go back to asking about your ideal exam type and what kind of contribution towards your degree for exams during COVID would be ideal for you.
Participant: So I think, obviously this would be specifically for medicine, I personally don't- I personally think that the contribution of the online exam should be kept the same. And perhaps in the future when things hopefully have settled down a little bit, then our clinical competencies are so reassessed or in like a future additional OSCE or something like that because I feel like we shouldn't- sorry your question was you asking about the sort of weighting right? Yeah, yeah, so I personally- my personal opinion is that we shouldn't increase the weighting of the online exam. Because I feel like that's not representative, alone. Some students do really well in the in written papers, but may- but there are also other students who excel in their clinical competencies, and so these two types of exams- clinical and written exams- really assess different types of students, which ultimately leads to different types of doctors and so I personally feel that this should be kept the same, the weighting. And the percentage that was missed out because of the OSCE cancelling should be sort of evaluated in the future and reassessed in the future.
Interviewer: Ok, thank you very much for that, that was really helpful. Is there anything else you'd like to add?
Participant: No I think-
Interviewer: Just in general about anything we discussed.
Participant: No, I don't think so. I think I've said pretty much everything.
Interviewer: Um, in that case, I think we're done with the interview. Thank you so so much for agreeing to do this and taking the time, it's been so helpful.
Participant: No problem. Are you stopping the recording now or?
Interviewer : Yeah, I'm stopping the recording now, if there's nothing else you want to add?
Participant: No, that's everything.
Interviewer: Ok yeah, I'll stop the recording.

Interview transcript 6

Interviewer: You should get a voice recording as soon as the interview is over and then you should get the transcript a few days later.
Participant: Ok
And when you get the transcript you have two weeks to withdraw any bits of the interview, or the whole interview if you want and to do that, you just have to email us about what you want to withdraw.
Participant: Ok
Interviewer: So you have the two week time period and throughout this interview feel free to stop me at any point if you don't wish to continue or if you want to take a break or if you have any questions or anything like that. Just feel free to stop me at any point. Um yeah, and before we start, do you have any questions?
Participant: No, I don't think so now.
Interviewer: OK, great, let's start then. So what were the changes to your exam?
Participant: Um so I was just doing like a multiple choice exam. Um, so and- so that was then put online, um to an open book exam, online, the same multiple choice, the same number of questions, and then the anatomy exam that we had was cancelled and the OSCE was cancelled as well.
Interviewer: OK
Participant: And in terms of the weighting of the exam, just became 100% of semester 2 for that year and they just put some of the anatomy and some of the OSCE into the exam- multiple choice that was open book.
Interviewer: Yeah.
Participant: And that was it, it wasn't too dramatic really, apart from the cancellations, obviously.
Interviewer: OK, so just summarizing. So you had previously you would have had one exam- multiple choice - and was that meant to be in a hall?
Participant: Yeah, yeah it would have been invigilated previously.
Interviewer: OK, and that has now turned into an online open book exam?
Participant: Yeah
Interviewer: OK, and was this exam supervised, unsupervised?
Participant: Unsupervised, yeah, they just- we got given an email address if we had any technical issues.
Interviewer: OK um, can you just take me through exactly how this you carried out this exam then? Just like a brief outline.
Participant: Uh, ok so, we got given an email 5 minutes before with a link to like a login page and then like, oh sorry, 30 minutes before we got given a link to a login page and then 5 minutes before the exam started we got given the password so we like enter our username. We got given like a password each, and then we just started the exam, like I think they gave us that kind of five minute leeway to like work out how to log in and stuff and then we had the usual exam time that we would have had to do the online exam and you could use Google, like you could use your notes and everything as well.
Interviewer: So did everyone have the exam at the same time or was there- you could choose?
Participant: Yeah, it was all at the same time, so they had to make it friendly for people who were overseas and had different time zones. So it was like midday to help them
Interviewer: OK and the timing stayed the same as it would have been before? So the-
Participant: No-
Interviewer: So how long the exam lasted.
Participant: Oh sorry yeah yeah, yeah
Interviewer: Ok
Participant: It was the same.

Interviewer: And um, so can you tell me how you felt about this change from this in hall assessment to now this online exam?
Participant: Uh, well I was initially kind of relieved because we obviously had those other two exams cancelled - the anatomy and the OSCE, so it was a lot less work [laughs]. But they kept giving us notifications that they knew that the exam was open book so that meant they'd probably make it harder, and so I was quite - like I was quite worried about like how hard it would be. Because when - you usually get to know the exam format in medical school, but then if they change it, you don't really know what to prepare for. And I was also quite worried about, like all the content from the anatomy and the OSCE that were cancelled. We're not doing them again until Year Four
Interviewer: Ok
Participant: So we won't have that knowledge and it will be a lot more to learn really.
Interviewer: Yeah, OK, any other comment about this change?
Participant: Um, I don't think so. I mean, there were so many other things going on at the time. Yeah, it was just- I don't think it was, it wasn't really unexpected and we usually- it was the same format really, so it wasn't too bad.
Interviewer: How did you feel about it being open book?
Participant: Um, I was kind of confused about how it would work if they weren't invigilating it and we could just use anything, but I did- it did kind of make me relax a bit more than I usually would with the exams because obviously I knew that I had to fall back- I tried as much as I could to just revise as I usually would, but there was like a voice in my head that was like "it's fine you've got- you can have your notes around you and things" So yeah.
Interviewer: Yeah I think that's understandable.
Participant: It's hard
Interviewer: Yeah. And, um your anatomy- yeah, so for the purposes of this study, we're not going to focus on the OSCE side of things, but let's talk about your anatomy exam. What would that have been like before the cancellation?
Participant: So it was going to be this - this exam- this exam period for me was semester two of 2nd year so, um, it was going to be all the content from first and second year anatomy, which was like quite a bit of dread for us and it was going to be like-
Interviewer: Yeah that's quite a lot
Participant: 30 stations.
Interviewer: Yeah
Participant: So yeah, I really was dreading it, but yeah, I'm concerned that obviously I'm missing out on that knowledge now because I haven't gone back to revise at all. Obviously it wouldn't be feasible with coronavirus to have us all like sat and moving around and things, so it wouldn't have worked but yeah.
Interviewer: And so how do you feel about the, the, that exam being cancelled and the questions now being *added* to the written exam?
Participant: I mean, I kind of expected it because they had to test the knowledge somehow and they won't be able to defer the anatomy exam into further years, they said. So they usually put a little bit of anatomy in anyway, and it made sense to kind of cover that a little bit. Yeah, it wasn't too bad to revise.
Interviewer: OK. Now in terms of waiting, you said that this open book exam counted towards 100% of your year. What was it before that?
Participant: Oh, I will have to check that very quickly.
Interviewer: It's fine
Participant: It was a mixture of - we do SSC, student selected components- so that, that also got cancelled and that was going to be part of it as well. Sorry, I'll just-
So previously, it would have been mixed in with the OSCE. It's kind of confusing the way they put it out. It's more as a whole year, so the exam was still being 100% of semester 2 but then a part of the

year the OSCE would have been included- the way they put it out is very confusing. Sorry I can't really give you a very good answer.
Interviewer: That's ok, don't worry about it, so it- so it would have been this exam, the OSCE and-
Participant: The SSE, it got made formative instead of summative, so that mark didn't count
Interviewer: Ok. How much would the SSC have counted before?
Participant: Um-
Interviewer: It's ok if you don't know.
Participant: It was 50% of the- of that module- umm
Interviewer: It's OK, don't worry, we don't have to go into the details of that, but just- so this SSC has now been turned into a formative, was that still carried out online? How was it carried out?
Participant: Yeah, I mean it would have been kind of- it was like a group project and you submitted like a kind of blog thing, um so we still submitted the blog and we just did like group meetings online and so it didn't change it too much.
Interviewer: Right so how do you feel about it being cancelled and most of this year being the open book exam?
Participant: I think it gave me more chance to kind of focus on the exam content. It would have been quite a lot of other things to focus on and the SSE was- kind of in my head is -one of the kind of less important things, so the fact it was cancelled gave me more time to just focus solely on the exam. And *obviously it was a bit of a stressful time for everyone. So then your mind wasn't entirely on the like the exam itself. You're a bit like thinking about lockdown and all that so, yeah, I think it was good that they gave us that change.*
Interviewer: Ok
Participant: Less pressure.
Interviewer: Ok, so, and you did mention something about you having to do your anatomy and OSCE exams in year 4.
Participant: Uh, the- yeah, so we intercalate in year 3 and then at the start of Year 4, they're planning on doing the OSCE. But yeah anatomy is just - I don't think it's going to go ahead at all.
Interviewer: Oh ok, so how do you feel about the OSCE now being in the start of Year 4?
Participant: Um, a bit stressed because we've- I- we've never done like an examined OSCE-
Interviewer: Yeah,
-before, so that will be the first time in Year Four which is, I mean, that's quite far into medical school so yeah, I mean, I'll just take it as it comes. It's a bit annoying, so we obviously practiced all the techniques and everything a lot before it got cancelled. So I have to like revisit it all.
Interviewer: Yeah.
Participant: But yeah we'll just- just take it as it comes, I think!
Interviewer: So you're not going to have another one of your anatomy exam then>
Participant: No.
Interviewer: OK, and how do you feel about that? That's just- I guess- basically cancelled completely.
Participant: Yeah yeah. It's a lot of- it's a lot of knowledge that could be very useful in the future and I'm sure it would have been- it would have been a lot of effort as well, so it's kind of like pros and cons: like it would have been good to know all the stuff, but also it kind of saves me from that stress as well. So two sides to it.
Interviewer: Definitely, yeah. Ok, let's move on to the next question, which is, what would have been your ideal exam if you had to choose during these circumstances? What would you have preferred to happen?
Participant: Ok, I think the way it happened to be honest was quite was fine with me. I mean I can't really complain- it was open book, we could use like our notes and Google, it was multiple choice, it really wasn't that taxing and it ended up being like the same, really the same level of difficulty that it usually would be. So I really think it- you couldn't really ask for much more because they've

obviously got to give us an exam in some format, they can't just completely cancel them. So I would say it's pretty good, yeah.
Interviewer: Yes, so you would have stuck to your open book online exam that was a summative.
Participant: Yeah
Interviewer: Yeah, and would you have stuck to cancelling your anatomy exam and your OSCE exam as well?
Participant: Well in the, in the circumstances I can't really see how they would have gone ahead anyway. Obviously if they, if they could have and coronavirus didn't exist, I would have preferred to actually have that experience. But yeah, yeah, I mean, I suppose they couldn't really have planned ahead.
Interviewer: Ok and say, again, like coronavirus didn't exist and like in the future, everything's back to normal. What would your ideal exam type be then? Would you still prefer online exam or would you go back to in hall exams? Or would you prefer something else?
Participant: I quite like the feeling of being around other people doing the exam at the same time. I think it gives you the right level, of kind of, being in the right mindset. When you're at home and you're just like sitting at adesk, you don't really understand the important- importance of the exam as much. Yeah, I think I would much prefer to be with other people doing an exam, but yeah, that's it, I mean. It's a bit of a cheat, but we always have multiple choice exams, in our kind of knowledge exam, so yeah.
Interviewer: Um, anything other than you guys, you guys - everyone being the same environment, anything else, any other reason you'd prefer an in hall assessment.
Participant: Probably because it's actually testing your knowledge and you feel like you actually have to learn the content. If you don't learn it, you're not going to be able to input it in the exam, whereas I knew if I didn't learn the content in this online exam then I could just find it in my notes, which was a bit of a cheat, yeah.
Interviewer: Yeah, any other points?
Interviewer: I don't think so, no, sorry.
Interviewer: That's ok. And what would you have preferred in terms of weighting changes?
Participant: Um…I think it was fair enough to make it 100% of semester two and maybe that SSC that got cancelled could have contributed a bit more because we'd already done the work by that point, so it seemed like a bit of a waste. But I think the way they handled it was fine, and I didn't mind it being 100% because I suppose it's the same as school exams. They're usually like 100% of your grade, so it's just the same thing really. I didn't mind it was 100% percent.
Interviewer: Yes, OK, ok. So let's move on to the next question, which may sound a bit similar. But what are the most important aspects to you of an exam for it to be satisfactory or for it to be a good exam? So when we talk about this, we're not really talking about the contents that the exam assesses, but more the exam itself.
Participant: OK, probably understanding the format of the exam, kind of being familiar with it. I was familiar with it, but if they'd all of a sudden said oh, it's going to be like essays or something, like short essays, I'd be like confused and yeah, not really sure what to do, so I think it's, yeah, important to know the kind of format and what you're going into. Kind of knowing the timing that you've got, like enough time to complete it and like being familiar with that and how much time you spend on each question. Um, like know the technology because I was quite scared that my wifi was gonna completely go or something [laughs]. So yeah, being able to rely on the equipment you're using so you can just focus on actually answering the questions rather than worrying on the outside, kind of influence. Making sure you have like quiet and all that, no interruptions.
Interviewer: Yeah, yeah, anything else?
Just knowing that, yeah, I'm up- carrying out your full potential. You're not ill or feeling under the weather or anything. Like if I'd had coronavirus, I'm not sure how they would have dealt with that. Yeah, particularly so. Yeah, I'm not sure.

Interviewer: Definitely yeah, thank you so much for that. That's really helpful. OK, next question is compared to before coronavirus and now with all the changes, how was your confidence going into the exam?
Participant: Um, I'd say I was a bit apprehensive because obviously I didn't know entirely how hard it was going to be, and I was a bit more confident as I knew it was open book so I knew I always had something to rely on, like my notes-my notes, but um yeah, I was a bit worried for the difficulty and also for – my sister had done her exam the week before and the Internet had actually cut out during the exam
Interviewer: Oh no, yeah
Participant: So I was, I was quite worried that the same thing was going to happen to me and we'd only been given an email address, like I think I would have preferred that they've given us a phone number, actually, like phone, if I had technical issues and stuff, but they just gave us an email address so I'm not sure how quickly they would have responded and they said they wouldn't give any additional time to people if they dropped out like for Internet.
Interviewer: Oh ok
Participant: So I was a little bit stressed.
Interviewer: Yeah I can imagine
Participant: Yeah, but I imagine it was the same for most people doing online exams – it just wasn't ideal. But I'm sure like if they did it in the future, they would hopefully have better systems in place.
Interviewer: Yeah, hopefully
Participant: I think I- yeah I was a little bit less confident as well because you don't have your peers around you. You know, you kind of chat nervously and kind of hype yourself up a bit, but when you're on your own, it's a bit different. It's not quite as- it doesn't feel the same. Yeah.
Interviewer: Yeah. Did you have any circumstances leading up to the exam that affected your teaching or your confidence level at all?
Participant: No, I don't think so. No
Interviewer: Ok
Participant: They'd given us some kind of- they'd given- they'd given us some pre-recorded lectures to fill in the lectures that we couldn't go to obviously, but for some they'd just given us like a PowerPoint with pictures and said oh we can't get a recording, so you're just going to have to follow that through, but this is examinable so it was a bit kind of vague and you weren't really sure how much you needed to know and.
Interviewer: Yeah, OK. Everything in terms of learning the content was fine for you then.
Participant: Yeah, yeah, I had no like special circumstances or anything.
Interviewer: OK, and did the fact that this exam counted towards 100% of your grade, did that affect your confidence level at all?
Participant: Um, not too much because I thought there was nothing really else they could do about it and I don't think I minded, yeah, because I knew it was open book. And yeah, no, it wasn't too bad.
Interviewer: OK. OK, that's, that's all the questions I had for you. Again, thank you so much for taking the time out to do this. Honestly, it's been so helpful for us.
Participant: That's OK, are you doing a research project on this? Or
Interviewer: Yeah, we're-
Participant: Yeah
Interviewer: Yeah, we're doing, we're still researching about students' opinions about all the changes and hopefully we'll be able to publish it after we collect all our data and then analyze them. So that's what we're hoping for.
Participant: OK, yeah, a lot of work for you, though, I imagine [laughs]
Interviewer: No, I mean we- yeah we're like halfway through, so we'll have to pull through.
Participant: Yeah [laughs] Aww.
Interviewer: Yeah again, yeah you will get the recording as soon as this interview is done. And you'll get the transcript in a couple of days. Do you have any questions at all for us?

Participant: No, I think that's everything, yeah.
Interviewer: Do you want to add anything that I may not have asked you or any other comments?
Participant: No, I think that was pretty much everything that needed to be covered here, yeah.
Interviewer: OK, OK, great. Thank you so much again for taking part.
Participant: That’s ok.
Interviewer: It's been really helpful. Thank you.
Participant: Don’t worry, good luck!
Interviewer: Thank you, bye
Participant: Bye.
